# Multiple freezing–melting pathways of high-density ice through ice XXI phase at room temperature

**DOI:** 10.1038/s41563-025-02364-x

**Published:** 2025-10-10

**Authors:** Yun-Hee Lee, Jin Kyun Kim, Yong-Jae Kim, Minju Kim, Yong Chan Cho, Rachel J. Husband, Cornelius Strohm, Emma Ehrenreich-Petersen, Konstantin Glazyrin, Torsten Laurus, Heinz Graafsma, Robert P. C. Bauer, Felix Lehmkühler, Karen Appel, Zuzana Konôpková, Minxue Tang, Anand Prashant Dwivedi, Jolanta Sztuck-Dambietz, Lisa Randolph, Khachiwan Buakor, Oliver Humphries, Carsten Baehtz, Tobias Eklund, Lisa Katharina Mohrbach, Anshuman Mondal, Hauke Marquardt, Earl Francis O’Bannon, Katrin Amann-Winkel, Choong-Shik Yoo, Ulf Zastrau, Hanns-Peter Liermann, Hiroki Nada, Geun Woo Lee

**Affiliations:** 1 Frontier of Extreme Physics, Space Metrology Group, Korea Research Institute of Standards and Science, Daejeon, Republic of Korea; 2 Lawrence Livermore National Laboratory, Livermore, CA USA; 3 Deutsches Elektronen-Synchrotron, Hamburg, Germany; 4 Freiberg Center for Water Research, Freiberg, Germany; 5 The Hamburg Centre for Ultrafast Imaging, Hamburg, Germany; 6 European XFEL, Schenefeld, Germany; 7 Helmholtz-Zentrum Dresden-Rossendorf, Dresden, Germany; 8 Max-Planck Institute for Polymer Research, Mainz, Germany; 9 Department of Physics, Johannes Gutenberg University Mainz, Mainz, Germany; 10 Institut für Mineralogie, Universität Münster, Münster, Germany; 11 Department of Earth Sciences, University of Oxford, Oxford, UK; 12 Department of Chemistry, Washington State University, Pullman, WA USA; 13 Graduate School of Engineering, Tottori University, Tottori, Japan; 14 Applied Measurement Science, University of Science and Technology (UST), Daejeon, Republic of Korea

**Keywords:** Phase transitions and critical phenomena, Structure of solids and liquids

## Abstract

Various metastable ice phases and their complicated transition pathways have been found by pressurization at low temperatures at which slow kinetics and high metastability are easily achieved. By contrast, such diversity is less expected at room or elevated temperatures. Here, using a combination of a dynamic diamond anvil cell and X-ray free electron laser techniques, we demonstrate that supercompressed water transforms into ice VI through multiple freezing–melting pathways at room temperature, hidden within the pressure region of ice VI. These multiple transition pathways occur via a metastable ice (more specifically, ice XXI with body-centred tetragonal structure ($I\bar{4}2d$)) discovered in this study and a metastable ice VII that exists within the pressure range of ice VI. We find that supercompressed water structurally evolves from high-density water to very-high-density water, causing multiple transition pathways. These findings provide an insight to find more metastable ice phases and their transition pathways at elevated temperatures.

## Abstract (web summary)

Multiple freezing–melting pathways of ice VI are found at room temperature, occurring via the ice XXI and ice VII phases. Supercompressed water structurally evolves from high-density water to very-high-density water.

## Main

Water (H_2_O), composed of only two elements, forms numerous polymorphic phases from ice I_h_ to ice XX (refs. ^1–6^) and four amorphous phases^7,8^. Understanding the formation and transition pathways of the diverse H_2_O phases has been of interest in high-pressure physics and the search for life in space and on icy moons^9–11^ for a century.

Generally, the abundant H_2_O phases result from the configurational changes of hydrogen-bond networks (HBNs), which are tuned by the interplay of increasing packing density and lowering bonding energy over a wide range of temperatures and pressures^12^. When ice is pressurized over its equilibrium phase boundary at low temperatures, slow kinetics prevent the immediate configurational changes of HBNs, allowing the ice to be trapped in higher-energy states with increased packing density and bonding energy. In this case, the ice cannot often transform directly into a stable phase by reducing the total free energy because of insufficient thermal energy available at low temperatures. Thus, to reduce the total Gibbs free energy at a little cost, the overpressurized ice phase explores various metastable states with slightly lower energies in the energy landscape by adjusting the HBN configurations. This process yields various metastable crystalline and amorphous ice phases^1,3–8^, leading to their complicated transition pathways. In addition to the slow kinetics, rapid pressurization can easily bring the ice to higher metastable states, altering transition pathways via unexpected metastable phases^13–16^.

By contrast, at elevated temperatures at which the H_2_O molecules in HBN are thermally activated, pressurized H_2_O exhibits fewer metastable phases and a relatively simple transition pathway. However, the instantaneous cooperative motion of H_2_O molecules in HBN can still be hampered at elevated temperatures, when highly compressed water crystallizes rapidly into dense and complicated hydrogen-bonded ice phases. For example, it has been reported that metastable ice VII (refs. ^17,18^) and high-density amorphous ice (HDA)^19,20^ are formed during the fast crystallization of highly overpressurized water at room temperature within the stable ice VI or ice VII pressure regime. Theoretical studies have shown that plastic ice VII with defective hydrogen bonds can form from supercompressed water (SW) at elevated temperatures^21–23^, due to the decoupling of rotational and translational ordering of H_2_O molecules on rapid crystallization^21^. In addition, the formation of metastable phases in SW reflects the similar local structure of SW and the metastable phases, which can lower the nucleation barrier for the metastable phases rather than for stable phases^17,21,24–30^. This implies that SW can structurally evolve further with pressure, influencing the phase selection and freezing–melting pathways. Thus, even at elevated temperatures, additional metastable ice phases and transition pathways can be found depending on the degree of metastability and transition rates.

In the present study, we discover at least five different freezing–melting pathways of ice VI at room temperature, which are hidden within the stable ice VI pressure regime. The combined technique with dynamic diamond anvil cell (dDAC) and X-ray free-electron laser (XFEL) reveals the multiple transition pathways emerging through two metastable phases of ice XXI and ice VII (ms-ice XXI and ms-ice VII, respectively). Moreover, molecular dynamics (MD) simulation shows that SW evolves from high-density water (HDW) to very-high-density water (VHDW). This structural evolution of SW plays a key role in the phase selection of the ms-ice XXI, ms-ice VII and ice VI phases and, thus, their complicated transition pathways.

## Results

### Five types of pressure–time curve in the freezing–melting process

**Fig. 1:**
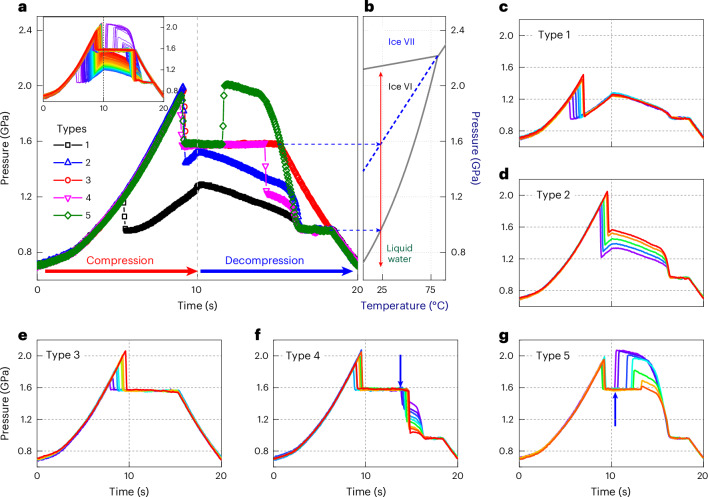
*P*–*t* curves during compression and decompression cycles. **a**, Five representative *P*–*t* curves are classified from the raw data for 1,000 dDAC cycles in the inset. **b**, Phase diagram of H_2_O; phase boundaries are depicted by solid lines and the metastable extension of the ice VII melting line is shown by the blue dotted line in the ice VI regime. The arrows from both pressure plateaus in the *P*–*t* curves meet with the coexistence pressures of liquid water and ice crystals. **c**–**g**, Typical *P*–*t* curves for type 1 (**c**), type 2 (**d**), type 3 (**e**), type 4 (**f**) and type 5 (**g**). The blue arrows in **f** and **g** indicate the second crystallization event from the mixed phase at 1.6 GPa. Standard deviation of pressure measurement is 0.003 GPa (ref. ^32^). Source data

By using a piezo-actuated dDAC operating at various (de)compression rates (0.001 to 120 GPa s^−1^ in this study), we repeatedly compress and decompress liquid water for 100 to 1,000 cycles at room temperature (see refs. ^31,32^ and Supplementary Figs. 1 and 2). During (de)compression, we simultaneously measure the pressure and optical image with time. The complicated pressure–time (*P*–*t*) curves (Fig. 1) reflect that the pressure-induced freezing–melting process of ice VI may not be as simple as generally expected. The *P*–*t* curves are categorized into five representative types, which happen randomly (Fig. 1 and Supplementary Figs. 1 and 2).

**Fig. 2:**
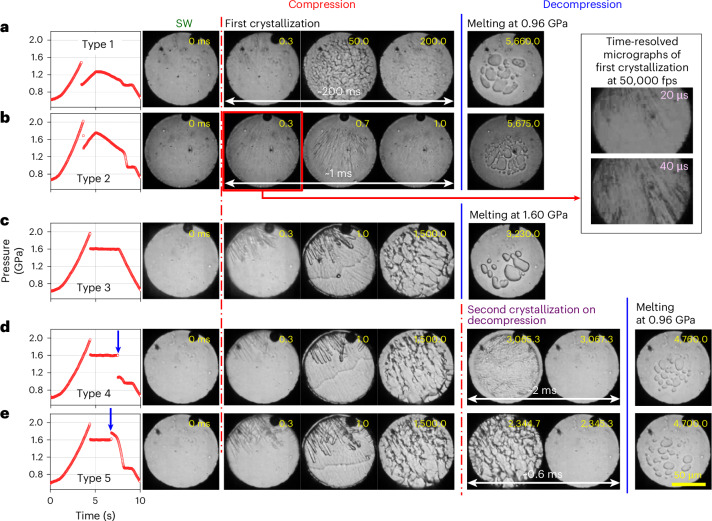
Optical images measured simultaneously with five types of *P*–*t* curve. The pressurization cycles are obtained within 10 s. **a**, Crystal growth is observed after the pressure drop and during the long melting plateau in type 1. **b**, First and second crystallizations are completed within 0.3 ms and 1 ms, respectively, in type 2. Inset: high-speed imaging at a rate of 50,000 fps, revealing that the first phase crystallizes within 40 μs (Supplementary Fig. 3). **c**, Mixture phase of ms-ice VII and water forms and melts away at the end of the pressure plateau at 1.6 GPa. **d**,**e**, Images of the second crystallization on decompression in type 4 (**d**) and type 5 (**e**), which occurs at 1.6 GPa (see the arrows in the *P*–*t* curves). Source data

Typical *P*–*t* curves for the freezing–melting process of ice VI, which is denoted as type 1, are shown in Fig. 1c; the pressure of SW suddenly drops to the equilibrium melting pressure of ice VI (0.96 GPa) at room temperature. Owing to the partial crystallization of SW, the remaining water crystallizes at 0.96 GPa, with a plateau during compression. On decompression, ice VI melts again, with a long plateau at 0.96 GPa. Optical images support this freezing–melting process (Fig. 2a). In type 1, all *P*–*t* curves overlap in the compression–decompression cycles after crystallization.

The type 2 pathway occurs when water is highly pressurized above 1.6 GPa; in this case, the pressure drops due to crystallization are always higher than 0.96 GPa and widely distributed within the pressure range of stable ice VI (Fig. 1d), unlike type 1. Interestingly, high-speed optical images reveal two rapid solidification events occurring during the sudden single pressure drop on compression (Fig. 2b and Supplementary Fig. 3), that is, fast crystal growth from SW within 20–40 μs and another subsequent growth with featureless crystal morphology from the outer border towards its center within approximately 1 ms.

The type 3 pathway exhibits a long plateau at 1.6 GPa after the first pressure drop (Fig. 1e), indicating the formation of a mixture of ms-ice VII and water in the stable ice VI regime^14^. At the end of the plateau at 1.6 GPa, the pressure decreases monotonically without the melting plateau at 0.96 GPa during decompression. This reflects the complete melting of ms-ice VII at 1.6 GPa, as confirmed by optical images (Fig. 2c).

The mixture of ms-ice VII and water at 1.6 GPa in type 3 occasionally transforms into another phase with a sudden pressure drop or jump during decompression (Fig. 1f,g, arrows), depending on the volume fraction of ms-ice VII in the mixture. When the volume fraction of water is larger than that of ms-ice VII at the end of the pressure plateau (1.6 GPa), volume shrinkage from water to ice VI is larger than volume expansion from ms-ice VII to ice VI, yielding the pressure drop. Pressure jump occurs when ms-ice VII has a larger volume fraction than water at the end of the pressure plateau (Supplementary Fig. 1). The resulting pressures after the sudden pressure drop and jump from 1.6 GPa are widely distributed within the pressure range of stable ice VI and then show slopes (or plateaus) with time during decompression. Subsequently, we observe another pressure drop to 0.96 GPa. These *P*–*t* curves with pressure drop and jump are denoted as types 4 and 5, respectively (Fig. 1f,g). The corresponding optical images (Fig. 2d,e) show that the first crystallization is identical to type 3 and the second crystallization, accompanied by a pressure drop or jump from 1.6 GPa, shows a featureless crystal morphology seen in type 2.

We identified the phases appearing in the five types of *P*–*t* curve as water, ice VI and ms-ice VII using synchrotron X-ray diffraction and micro-Raman spectroscopic studies (Supplementary Figs. 4 and 5). Thus, the freezing–melting process appears to occur through three different pathways: SW→ice VI→water (type 1), SW→ms-ice VII→water (type 3) and SW→ms-ice VII→ice VI→water (types 4 and 5). However, the rapid two-step transition in type 2 (Fig. 2b) was not directly identified because of the insufficient time resolution in synchrotron XRD and Raman experiments.

### XFEL time-resolved study of crystallization on SW

**Fig. 3:**
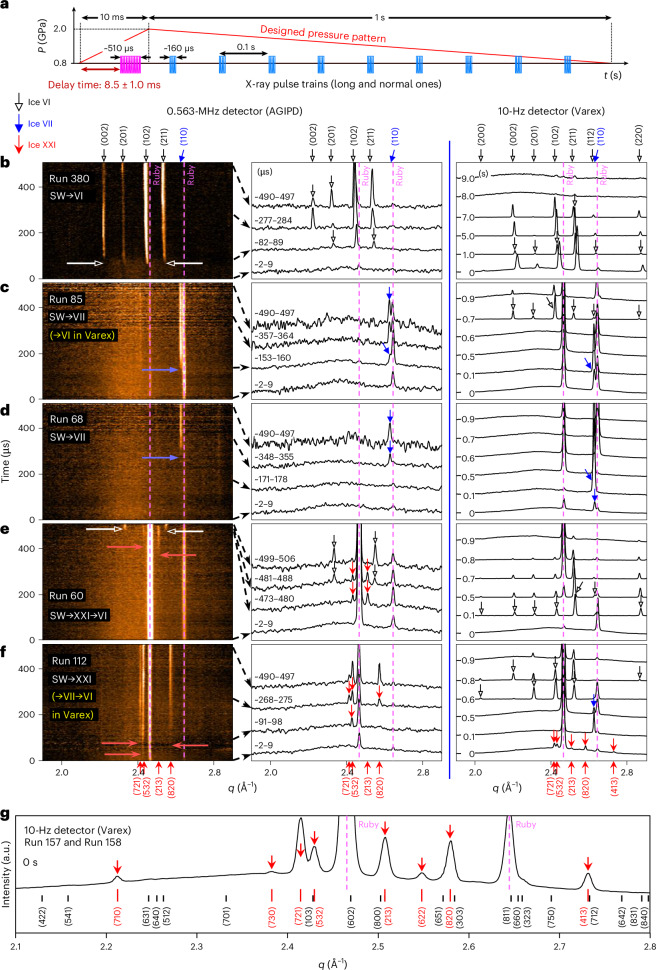
Time-resolved X-ray diffraction patterns detecting the crystallization phases in XFEL experiments. **a**, X-ray pulse trains synchronized with the pressure profile. During compression for 10 ms, a long X-ray pulse train with a duration of ~510 µs is applied at 8.5 ± 1.0 ms to detect the crystallization events. The maximum compression reaches ~2 GPa. For 1-s decompression, 9 consecutive X-ray pulse trains with a duration of ~160 µs are applied. **b**–**f**, Detection of phases for the first crystallization from SW and their transitions to the stable ice VI phase during (de)compression. Shown are representative streak images of SW→VI (**b**; Run 380), SW→VII with transition to VI in Varex (**c**; Run 85), SW→VII (**d**; Run 68), SW→XXI→VI (**e**; Run 60) and SW→XXI with transition to VII and VI in Varex (**f**; Run 112). Left: diffraction patterns recorded for the crystallization events during 10-ms compression. The arrows mark the appearance of crystal peaks. Subsequent transitions observed with Varex, where ms-ice VII and ms-ice XXI of **c** and **f** are converted to stable ice VI, are shown in the parentheses. Middle: diffraction peaks of the AGIPD data averaged over 7 µs for clarity. Right: diffraction patterns obtained during decompression using the Varex detector. **g**, Representative diffraction peaks of the ice XXI phase obtained by the Varex detector during crystallization are indexed with the best-fitting body-centred tetragonal structure ($I\bar{4}2d$) and lattice parameters *a* = *b* = 20.085 Å and *c* = 7.828 Å (details in Supplementary Figs. 8a, 9c and 10). In **e** and **f**, the (710), (730) and (622) peaks of the ice XXI phase are missed due to the occurrence of preferred orientation in the initial fast crystallization (Supplementary Fig. 8b). Source data

To resolve the rapid two-step transition that occurs within tens of microseconds in type 2, we utilized a megahertz pulse X-ray source during dynamic pressurization (High Energy Density (HED) beamline at the EuXFEL)^33,34^. After careful synchronization of the crystallization events and X-ray pulse trains (Fig. 3a), we successfully captured the ice crystallization events with two fast detectors: a pulse-resolved adaptive gain integrating pixel detector (AGIPD) with a detection rate of 0.56 MHz (ref. ^35^) and a train-resolved Varex detector with a rate of 10 Hz (details are given in Fig. 3a, Methods and Supplementary Fig. 7a). We found that the five types of *P*–*t* curve were still maintained at a fast compression rate of 10 ms (Supplementary Fig. 6). Thus, the pathways of the freezing–melting process were elucidated in detail by the XFEL experiment (Fig. 3).

Crystallization of ice VI and ms-ice VII in SW is detected in Fig. 3b,c, respectively. On decompression, the broad diffuse scattering intensity with crystalline peaks in the Varex detector reflects the coexistence of liquid water with ms-ice VII (0.0–0.6 s; Fig. 3c). Then, ice VI appears from the mixture phase of ms-ice VII and water at 0.7 s on decompression (Fig. 3c), corresponding to type 4 in the *P*–*t* curves. In Fig. 3d, the ms-ice VII coexisting with water melts completely and no other crystal phases appear during decompression, corresponding to type 3 in the *P*–*t* curves.

In particular, we discover an ice phase that directly forms from SW (Fig. 3e–g and Supplementary Fig. 7b). The XRD pattern of the discovered ice phase does not match with the ice I_h_ to ice XX phases including the metastable phases^36–38^ in the ice VI regime in experiments and three calculated phases in theoretical studies (Supplementary Fig. 8a). This ice phase has a body-centred tetragonal structure ($I\bar{4}2d$) with a large unit cell (*a* = *b* = 20.197 Å and *c* = 7.891 Å) at approximately 1.6 GPa, which we call ice XXI. The large unit cell contains 152 water molecules, resulting in a density of 1.413 g cm^−3^ (see the refinement in Supplementary Figs. 9c and 10 and Supplementary Tables 1 and 2). Here ms-ice XXI directly transforms into ice VI on compression (Fig. 3e). This behavior is consistent with the type 2 crystallization sequence shown in Fig. 2b. Remarkably, the ice phase transforms into not only ice VI (Fig. 3e) but also ms-ice VII (Fig. 3f and Supplementary Fig. 7b). It should be noted that the reverse transition (from ms-ice VII to ice XXI) has not been observed in this study, even though both metastable phases can form directly from SW. This suggests that the ice phase certainly has a higher Gibbs free energy than ms-ice VII at room temperature, but their values may be fairly close to each other. Furthermore, we recognize that only ice XXI transforms into ms-ice VII, whereas the remaining water does not (see the AGIPD and Varex data at 0–0.1 s; Supplementary Fig. 7b). By contrast, both water and ms-ice VII in the mixture phase transform into ice VI (indicated by the lack of diffuse scattering intensity in the Varex data at 0.7 and 0.6 s in Fig. 3c,f, respectively, and Supplementary Fig. 7b).

Accordingly, within the stable ice VI pressure regime at room temperature, the XFEL experiment reveals the ice XXI phase and the hidden pathways of crystallization–melting transition that emerge through the two metastable intermediate phases (MIPs; that is, ms-ice VII and ms-ice XXI). Thus, the freezing–melting process can take at least five different pathways according to the time-resolved observation: (1) SW→ice VI→water, (2) SW→ms-ice VII→water, (3) SW→ms-ice VII→ice VI→water, (4) SW→ms-ice XXI→ice VI→water and (5) SW→ms-ice XXI→ms-ice VII→ice VI→water.

## Discussion

### Multiple crystallization pathways from two forms of HDW

Ostwald recognized that MIPs often form from a metastable liquid before the occurrence of a thermodynamically stable phase, called Ostwald’s step rule^39^. Owing to the appearance of MIPs, crystallization occurs through multiple steps and multiple pathways, as we observe in this study. Ostwald’s step rule has been explained by three hypotheses: structural similarity between liquids and MIPs^40^, kinetic competition of nucleation and growth rates between the MIPs and stable phase^41^ and minimizing entropy production^42^. Therefore, here we discuss the multiple crystallization pathways by considering the structure of SW and the stability and competing kinetics of the MIPs^43^.

**Fig. 4:**
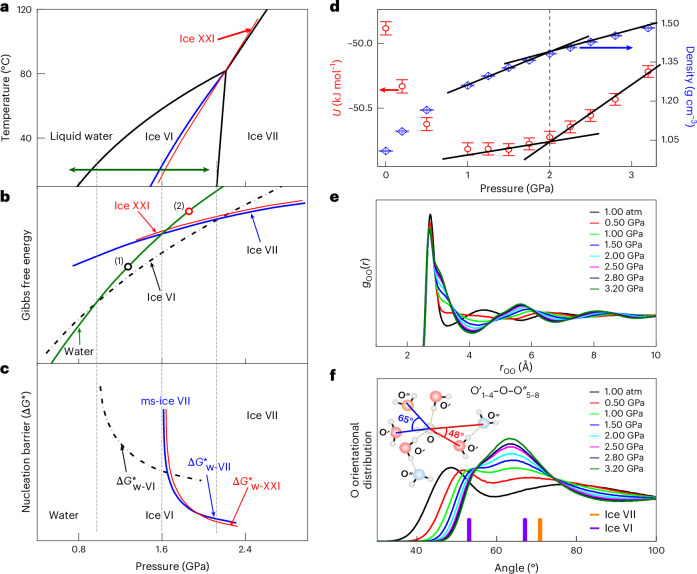
Phase stability of ice phases and MD simulation results. **a**, Phase diagram of H_2_O. **b**,**c**, Schematic of the Gibbs free energy (**b**) and nucleation barriers (**c**) for the four phases of water, ice VI, ms-ice VII and ms-ice XXI. The red lines in **a**–**c** denote the ice XXI phase, which are presumed, based on experimental observations (see details in the main text). **d**–**f**, Simulation results of SW are obtained using the SPCfw potential. **d**, Potential energy and density versus pressure. The black solid lines are guides for the eye. Data are presented as mean values, with the error bars indicating the s.d. calculated from 500 data points sampled every 10 ps over trajectories of 15–20 ns. **e**, PDFs of O–O atoms. **f**, ADFs of oxygen atoms in the first-nearest and second-nearest neighbours, O′_1–4_ and O″_5–8_, where O′_1–4_ and O″_5–8_ are defined as the 1st–4th and 5th–8th neighbouring O atoms around the O atom of the central water molecule, respectively. Source data

The nucleation competition between the crystal phases can be understood by their phase stabilities, as inferred above. Although SW transforms into the ice VI phase that has the lowest Gibbs free energy below 1.6 GPa (marked by (1) in Fig. 4b), it can transform into two MIPs over 1.6 GPa as well as stable ice VI (marked by (2) in Fig. 4b). The formation of ice XXI in SW and the one-way transition from ms-ice XXI to ms-ice VII over 1.6 GPa reflect the smaller nucleation barrier and higher Gibbs free energy of ice XXI compared with ms-ice VII (Fig. 4b,c). In addition, the emergence of both MIPs from SW means that the Gibbs free energies of the two MIPs are fairly close (Fig. 4b).

According to classical nucleation theory^17,43^, the nucleation barrier is given by ${\Delta G}^{\mathrm{*}}=\frac{16{\rm{\pi }}{\sigma }_{\mathrm{sl}}^{3}}{3{({\Delta g}_{{\mathrm{v}}}^{{\mathrm{s}}\mathrm{-}{\mathrm{l}}})}^{2}}$, where *σ*_sl_ is the crystal–liquid interfacial free energy and ${\Delta}g_{\rm{v}}^{\,{\rm{s}}-{\rm{l}}}$ is the difference in volume Gibbs free energy between crystal and liquid (that is, ${g}_{\rm{v}}^{{\,}\rm{s}}-{g}_{\rm{v}}^{\rm{l}}$) that provides a driving force for crystallization. Because ${\Delta}g_{\mathrm{v}}^{{\mathrm{VII}}-{\mathrm{W}}}$ is smaller than ${\Delta} g_{\mathrm{v}}^{{\mathrm{VI}}-{\mathrm{W}}}$ in the range of 1.6–2.2 GPa (Fig. 4b), the formation of ms-ice VII suggests that *σ*_sl_ for ms-ice VII should be much smaller than that for ice VI to compensate for the smaller ${\Delta g}_\mathrm{v}^\mathrm{VII-W}$ and, thus, to yield a smaller nucleation barrier for ms-ice VII than for ice VI (ref. ^17^). Similarly, ms-ice XXI should have a smaller *σ*_sl_ than ms-ice VII, whereas competitive nucleation of the two MIPs also reflects their similar *σ*_sl_ values. Thus, the order of the interfacial free energy might be ${\sigma }_{\mathrm{sl}}^{\mathrm{XXI}} < {\sigma }_{\mathrm{sl}}^{\mathrm{VII}}\ll {\sigma}_{\mathrm{sl}}^{\mathrm{VI}}$ above 1.6 GPa.

From a microscopic viewpoint, *σ*_sl_ depicts the degree of structural similarity between the crystal and liquid, which is given by the difference in configurational entropy in both phases^44^. In previous studies^17,25^, the local order of HDW at 1 GPa was characterized as a body-centred-cubic-like structure, which is similar to the local order of ice VII, explaining the formation of ms-ice VII in SW^17^. However, if ice-VII-like local orders in SW prevail or strengthen with increasing pressure, it cannot explain the emergence of ice XXI over 1.6 GPa. This implies that the local structure of HDW should evolve further with pressure and become more similar to that of ice XXI than that of ms-ice VII, yielding ${\sigma }_\mathrm{sl}^\mathrm{XXI} < {\sigma }_\mathrm{sl}^\mathrm{VII}$.

The structural evolution of SW was investigated by MD simulation with simple point-charge flexible water (SPCfw)^45^ and TIP4P/Ice^46^ models. The simulation results using the SPCfw model reveal two different states of SW in potential energy and density above and below 2.0 GPa (Fig. 4d). Similar results were obtained using the TIP4P/Ice model (Supplementary Fig. 11). The equilibrium melting pressure of ice VI is higher by 0.46 GPa in the TIP4P/Ice model than in the experiment^31^. Thus, the pressure (2.0 GPa) reflecting the change in the properties in MD simulations roughly corresponds to the experimental pressure of 1.6 GPa.

In addition to the change in potential energy and density, pair distribution functions (PDFs; *g*(*r*)) of oxygen–oxygen (O–O) pairs also vary with pressure, reflecting the structural evolution of SW (Fig. 4e). Low-density water at 1 atm changes to HDW under pressure. With further compression, HDW shows distinctly enhanced shoulders in the first and second peaks at approximately 3.3 and 4.8 Å, respectively (Fig. 4e). This behavior has been observed when HDA transforms into very-high-density amorphous ice (VHDA)^47,48^. In the PDFs of O–O pairs, changes in the peak height and position reflect the distortion of the tetrahedral order in HBN with pressure. However, a relatively small variation with pressure was observed in the PDF of H–O pairs (Supplementary Fig. 12a).

Angle distribution functions (ADFs) of oxygen atoms between the first- and second-nearest neighbours (O′_1–4_–O–O″_5–8_) also exhibit the structural evolution of SW with pressure (Fig. 4f); between 1 and 2 GPa, two peaks of ADF around 53° and 65°, which are similarly observed in ice VI, exchange their relative intensities with pressure and then merge over 2 GPa, resembling ice VII. This behavior was also observed in a previous report^25^. On the other hand, the ADF of O–H–O_2_^H^ shows relatively small changes over 1 GPa and resembles somewhat between ice-VI-like and ice-VII-like ADFs with increasing pressure (Supplementary Figs. 11h and 12b). Here O_2_^H^ is the O atom of the water molecule that is included in a sphere with a radius of 3.5 Å from the O atom of the central molecule.

Accordingly, the changes observed in Fig. 4d–f reflect the structural evolution of SW from HDW to VHDW by distorting and rearranging the tetrahedral structures of HBN during supercompression, further reducing ${\sigma }_{\mathrm{sl}}^{\mathrm{XXI}}$ rather than ${\sigma }_{\mathrm{sl}}^{\mathrm{VII}}$, and facilitating the formation of ice XXI above 1.6 GPa observed in experiments.

A recent theoretical study^28^ suggested that VHDA transforms into metastable plastic ice VII and ice Y phases before the formation of stable ice VII during crystallization at room temperature. In addition, it was reported that dense liquid water is structurally similar to VHDA rather than ice VII, but differs from simple liquid (or hard-sphere-like liquid), with a randomly packed structure as the pressure and temperature increase to 6.5 GPa and 670 K, respectively^49^. These examples suggest that unlike HDA and HDW, the local structure of VHDA differs from that of ice VII. Thus, on the basis of the changes in PDF and ADF, as well as the formation of ms-ice XXI rather than ms-ice VII, VHDW can be considered a counterpart of VHDA. This fills the gap between VHDA and simple liquid at high pressures and temperatures. Furthermore, it has been reported that even at the molecular level, the symmetry change in the local order due to distortion can notably influence the crystallization pathways^50^. Therefore, this study demonstrates that the structural evolution of local order in SW from HDW to VHDW can govern the phase selection of MIPs and lead to multiple pathways of the freezing–melting process at room temperature, which is consistent with Ostwald’s step rule^39^.

In summary, controlling the metastability of SW reveals hidden multiple freezing–melting pathways of ice VI at room temperature, which occur through the metastable ice XXI phase and ms-ice VII in the stable ice VI pressure range. The crystal structure of ice XXI was identified as a body-centred tetragonal structure ($I\bar{4}2d$). This study accelerates the search for more metastable phases and transition pathways in H_2_O and aqueous solutions at high pressures and temperatures, consistent with theoretical predictions^21–24,26–30^. The present results will be useful for the development of precise atomistic potential models of H_2_O and water-containing substances (for example, salty water and protein solutions) and will provide insights into the exploration and design of new functional materials manipulated by transition pathways in many other materials systems.

## Methods

### Dynamic crystallization experiment with a piezo-actuated dDAC

We perform compression and decompression by using a piezo-actuated, backward-type dDAC, which can be operated with various (de)compression rates of 0.001–120 GPa s^−1^ in this study. Combination of high-speed camera, two Raman spectrometers and Fabry–Pérot interferometer with the dDAC enables the simultaneous measurements of sample images, pressure, volume and micro-Raman spectra during dDAC operation, called the real-time event monitoring system^32^. Deionized water (resistivity, 18.2 MΩ cm) is loaded into a stainless steel gasket hole with a diameter of 100–120 μm and a thickness of 30–50 μm. Liquid water is dynamically pressurized from 0.6 to 2.0 GPa crossing the equilibrium pressure of water-ice VI—0.96 GPa at room temperature—so as to investigate the freezing and melting processes. The dDAC increases and decreases the pressure repeatedly and precisely according to a triangular voltage function. During the compression–decompression cycle, pressure is measured from ruby fluorescence with short exposure times ranging from 1.6 to 100 ms and optical images are taken at a sampling rate of 3,000–50,000 fps. For the Raman scattering measurement, an exposure time of 2 s is used for (de)compression cycles of 200 s.

### Time-resolved X-ray diffraction experiment

The H_2_O phases appearing along the *P*–*t* curves during dDAC operation are identified by synchrotron X-ray diffraction facilities with high-energy beams (18 keV, 1C-XRS in PAL and 25.6 keV, P02.2 in PETRA III at DESY). By using an in-line ruby pressure measurement system installed at the synchrotron X-ray facilities^32^, both diffraction and pressure signals of the H_2_O phases are measured for every 100 ms during dynamic pressurization. The scattering signals recorded in a Lambda detector^51^ are processed with the DIOPTAS programme^52^. To resolve the two crystallization events in type 2, we combined XFEL (18.02 keV, HED beamline in EuXFEL) and dDAC (Supplementary Fig. 7a). The HED beamline provides a hybrid X-ray injection mode consisting of long- and normal-pulse trains; the long and short X-ray pulse trains persist for 510 and 160 μs, respectively, and the interval between the pulses is 1.78 μs (refs. ^33,34^). To synchronize the crystallization events with the 510-μs pulse train effectively, we modify the pressure pattern into an asymmetric triangular function with a short cycle which has 10-ms compression and 1-s decompression (Fig. 3a). X-ray diffraction patterns are recorded simultaneously in the AGIPD^35^ and Varex detectors corresponding to the long and short X-ray pulse trains. The repetition rates of both AGIPD and Varex detectors are 0.56 MHz and 10 Hz, respectively.

### MD of SW

MD simulations are performed to study the structural changes in SW from 1 atm to 3.2 GPa. We use two potential models of SPCfw^45^ and TIP4P/Ice^46^. The latter is a model of rigid H_2_O for which the phase diagram in the high-pressure region has been known, whereas the former is a model of flexible H_2_O for which the phase diagram in the high-pressure region is not known. Specifically, the former allows deviations of the H–O–H angle and O–H length of the water molecule from their equilibrium values caused by the formation of distorted hydrogen bonds, like in the structure of ice VI. Both models yield a similar trend of water properties with pressure, which qualitatively coincides with the experiment. The simulation system is a rectangular parallelepiped having 2,880 water molecules. Periodic boundary conditions are imposed in the *x*, *y* and *z* directions. Temperature is maintained at 298 K using a Berendsen thermostat with a thermal bath constant of 0.1 ps, and the pressure is kept constant using the Berendsen barostat with a pressure bath constant of 2.0 ps (ref. ^53^). Computations are carried out using the leap-frog algorithm with a time step of 0.5 fs for the SPCfw model and 1.0 fs for the TIP4P/Ice model. The total run time is 20 ns. The long-range Coulomb interaction is calculated using the Ewald method. The Lennard–Jones interaction acting on the O atom of the water molecules is truncated at an intermolecular distance of 1.0 nm. MD simulations are performed with DL_POLY_2.20 (ref. ^54^).

## Online content

Any methods, additional references, Nature Portfolio reporting summaries, source data, extended data, supplementary information, acknowledgements, peer review information; details of author contributions and competing interests; and statements of data and code availability are available at 10.1038/s41563-025-02364-x.

## Supplementary information

Supplementary Information Supplementary Figs. 1–12 and Tables 1 and 2.

Supplementary Data 1 Crystallographic information file for ice XXI.

## Source data

Source Data Fig. 1 Source data for Fig. 1.

Source Data Fig. 2 Source data for Fig. 2.

Source Data Fig. 3 Source data for Fig. 3.

Source Data Fig. 4 Source data for Fig. 4.

## Data Availability

Raw data recorded for the experiment at the EuXFEL are available at 10.22003/XFEL.EU-DATA-003379-00. X-ray crystallographic coordinates of water molecules in ice XXI have been deposited at the Cambridge Crystallographic Data Centre (CCDC) under deposition number 2478372. The crystallographic information file (CIF) of the ice XXI structure has been deposited in the Crystallography Open Database (COD) under COD ID 3000611. Source data are provided with this paper.
