# Supplementary material for: Multiple freezing–melting pathways of high-density ice through ice XXI phase at room temperature
Source: Nat Mater. 2025 Oct 10;25(2):302–9. doi: 10.1038/s41563-025-02364-x (PMC12867761; doi:10.1038/s41563-025-02364-x)
Supplement: Supplementary file 1 — Supplementary Figs. 1–12 and Tables 1 and 2. [file 41563_2025_2364_MOESM1_ESM.pdf]

nature materials

Supplementary information

https://doi.org/10.1038/s41563-025-02364-x

Multiple freezing–melting pathways of high-density ice through ice XXI phase at room temperature

In the format provided by the authors and unedited

Table of Contents

1. Pressure-time curves of freezing and melting processes during compression and decompression; cases of Type 1 and Type 2

Supplementary Figure 1. Schematic diagram of five types of pressure-time curves describing freezing-melting pathways of H_2O during compression and decompression.

2. Pressure-time curves for the cases of Type 3, Type 4, and Type 5

3. Massive pressure-time curves under dynamic pressurization

Supplementary Figure 2. Continuous compression and decompression cycles with one dDAC containing H_2O sample.

4. Time-resolved optical images of two crystallization events in Type 2

Supplementary Figure 3. Time-resolved micrographs of the crystallization events in Type 2.

5. In-situ synchrotron X-ray scattering experiment along the pressure-time curves

Supplementary Figure 4. X-ray diffraction results recorded simultaneously with pressure.

6. In-situ micro-Raman spectroscopic study along the pressure-time curves

Supplementary Figure 5. Phase identification with Raman spectra along the five types of P-t curve on crystallization-melting process.

7. Synchronization of the X-ray pulse trains and crystallization events during rapid compression in XFEL experiment

Supplementary Figure 6. Occurrence of crystallization during compression with different cycle periods.

8. Installation of dDAC in XFEL and event-synchronizing scheme

Supplementary Figure 7. dDAC setup for XFEL experiments and time-resolved diffraction data on crystallization events.

9. Comparison of the discovered ice XXI with 24 known ice phases

Supplementary Figure 8. Comparison of X-ray diffraction patterns of the discovered ice with 24 stable and metastable ice phases.

10. Structural identification of ice XXI based on single-crystal X-ray diffraction study

Supplementary Figure 9. A single crystal of ice XXI phase grown in the dDAC and representative XRD spot pattern indexed with the body-centred tetragonal structure.

Supplementary Figure 10. Crystal structure of ice XXI.

Supplementary Table 1. Crystallographic data collection and structural refinement for the merged diffraction data of single-crystal ice XXI under 1.6 GPa and room temperature.

Supplementary Table 2. Structural parameters of ice XXI under 1.6 GPa and room temperature.

11. Molecular dynamics study of supercompressed water by TIP4P/ICE model

Supplementary Figure 11. Simulation results with TIP4P/ICE model.

12. Pair distribution functions (PDFs) of O-H pairs and angle distribution functions (ADFs) from SPCfw model

Supplementary Figure 12. Changes in H-O pairs with pressure simulated with SPCfw model.

1. Pressure-time curves of freezing and melting processes during compression and decompression; cases of Type 1 and Type 2

When liquid water is compressed by a dynamic diamond anvil cell (dDAC), pressure bypasses the freezing pressure of ice and continuously increases. The liquid water that is supercompressed transforms into ice VI over 0.96 GPa at room temperature. Since the sudden transition from supercompressed water to ice VI is accompanied with density increase, pressure drops rapidly to 0.96 GPa where water and ice VI coexist at room temperature. By continuous compression, liquid water coexisting with the ice that crystallized with the pressure drop continuously transforms into ice VI with volume shrinkage. Since the external dDAC compression is nullified by the continuous volume exchange between water and ice VI, no pressure change occurs with time and finally this forms the pressure plateau observed in Supplementary Fig. 1. Once this crystallization process is completed, pressure increases upon further compression. Upon decompression, ice VI melts again at 0.96 GPa and forms a long plateau until the melting is completed. This is Type 1 pathway of pressure-time curves.

In case of Type 2, water is highly supercompressed over 1.6 GPa and crystallized with the pressure-drop. Since the volume of supercompressed water is already significantly reduced over 1.6 GPa, formation of dense ice VI phase cannot reduce the pressure to 0.96 GPa and makes pressure higher than 0.96 GPa. Then pressure increases and decreases during compression and decompression, respectively. Finally, we see the melting plateau at 0.96 GPa again upon decompression. This behavior is also observed in temperature-time curve, called hypercooling[55].

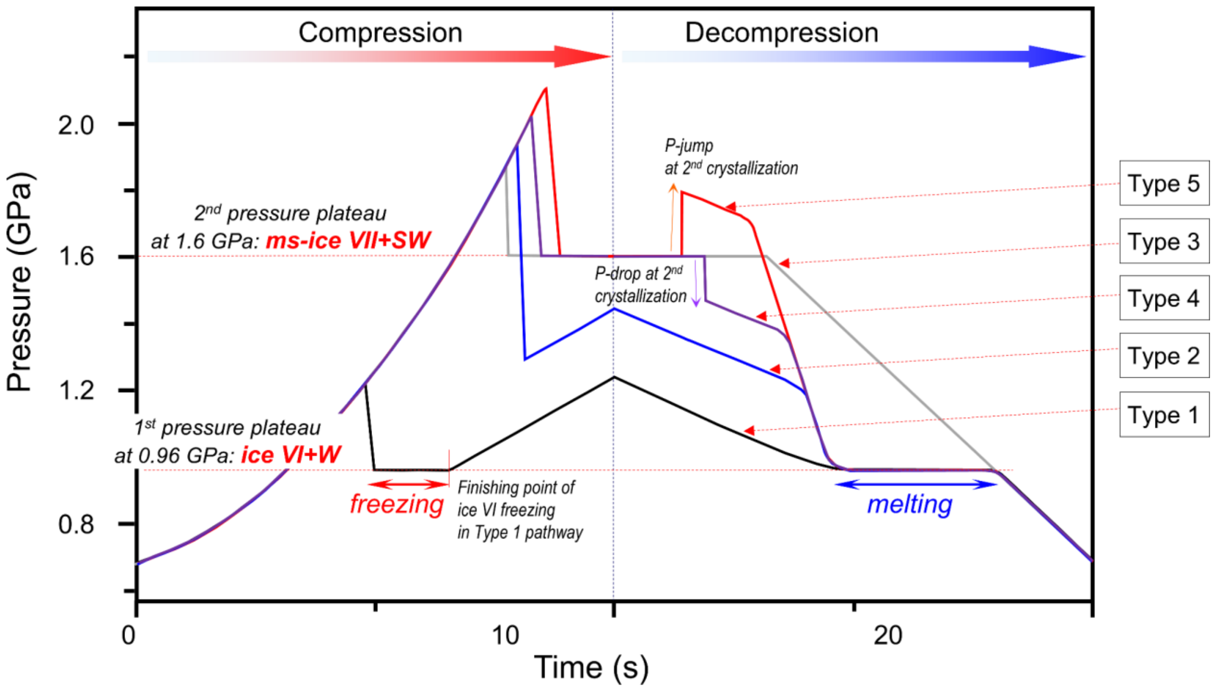

Supplementary Figure 1. Schematic diagram of five types of pressure-time curves describing freezing-melting pathways of H_2O during compression and decompression.

2. Pressure-time curves for the cases of Type 3, Type 4, and Type 5

When water is compressed over 1.6 GPa, crystallization causes pressure drop to 1.6 GPa, followed by a long plateau on (de)compression. At the end of this plateau, the pressure decreases monotonically upon decompression. This is the Type 3. As the pressure plateau at 0.96 GPa due to phase exchange between water and ice VI in Type 1, the plateau at 1.6 GPa means that two phases coexist and exchange their volume. In this case, the coexisting phases are metastable ice VII (ms-ice VII) and water, since the melting line of ice VII extends to 1.6 GPa in the ice VI phase field at room temperature. Therefore, the supercompressed water (SW) over 1.6 GPa transforms into the mixture of ms-ice VII and water on compression. On decompression, the ms-ice VII fully melts and only water remains at the end of the plateau. No observation of ice VI melting at 0.96 GPa supports this pathway.

Since both ice VII and water at 1.6 GPa are still metastable comparing to ice VI, they can transform into another metastable phase or stable one. Type 4 and Type 5 exhibit such cases, accompanied by pressure change. The crystallization behavior from SW is same as Type 3 on compression. However, the second crystallization from the mixture of ms-ice VII and water at 1.6 GPa leads to pressure-drop and pressure-jump. The pressure-drop and -jump in Types 4 and 5, respectively, are determined by the volume fraction of ms-ice VII phase and

water at 1.6 GPa. In detail, if water has larger volume fraction than ms-ice VII at the end of plateau at 1.6 GPa, the transition from the mixture to ice VI shows pressure-drop due to the significant contribution of volume shrinkage from water to ice VI than that of volume expansion from ms-ice VII to ice VI. Visa versa, if ms-ice VII has larger volume fraction than that of water at the end of plateau at 1.6 GPa, the transition from the mixture to ice VI gives pressure-jump since the contribution of volume expansion from ms-ice VII to ice VI is more significant than that of volume shrinkage from water to ice VI. Thus, the pressure-jump and -drop at the 2nd transition time is determined by the volume fraction of ms-ice VII and water at 1.6 GPa.

3. Massive pressure-time curves under dynamic pressurization

Liquid water is compressed and decompressed from 0.6 GPa to 2.0 GPa more than 100-1000 times by using dDAC. Supplementary Figs. 2a,b show a series of P-t curves from the repeating compression and decompression with 1000 cycles. Each cycle of the compression-decompression takes 20 s. Due to the quiescent pressurization, liquid water can be highly over-pressurized up to 1.8-2.0 GPa. The supercompressed water (SW) over 0.96 GPa shows the five representative types of P-t curves due to the freezing-melting process during compression and decompression (Fig. 1, and Supplementary Figs. 2a,b). It should be noted that the five types of P-t curves happen randomly (Supplementary Figs. 2a,b). In addition, the maximum pressure of the SW before crystallization exhibit randomness during the 1000 times operation in Supplementary Fig. 2c. The gasket hole (i.e., sample chamber) maintains almost its initial shape without serious deformation even after the 1000 cycles, although some debris from the gasket wall appear as the number of cycles increases (Supplementary Fig. 2d).

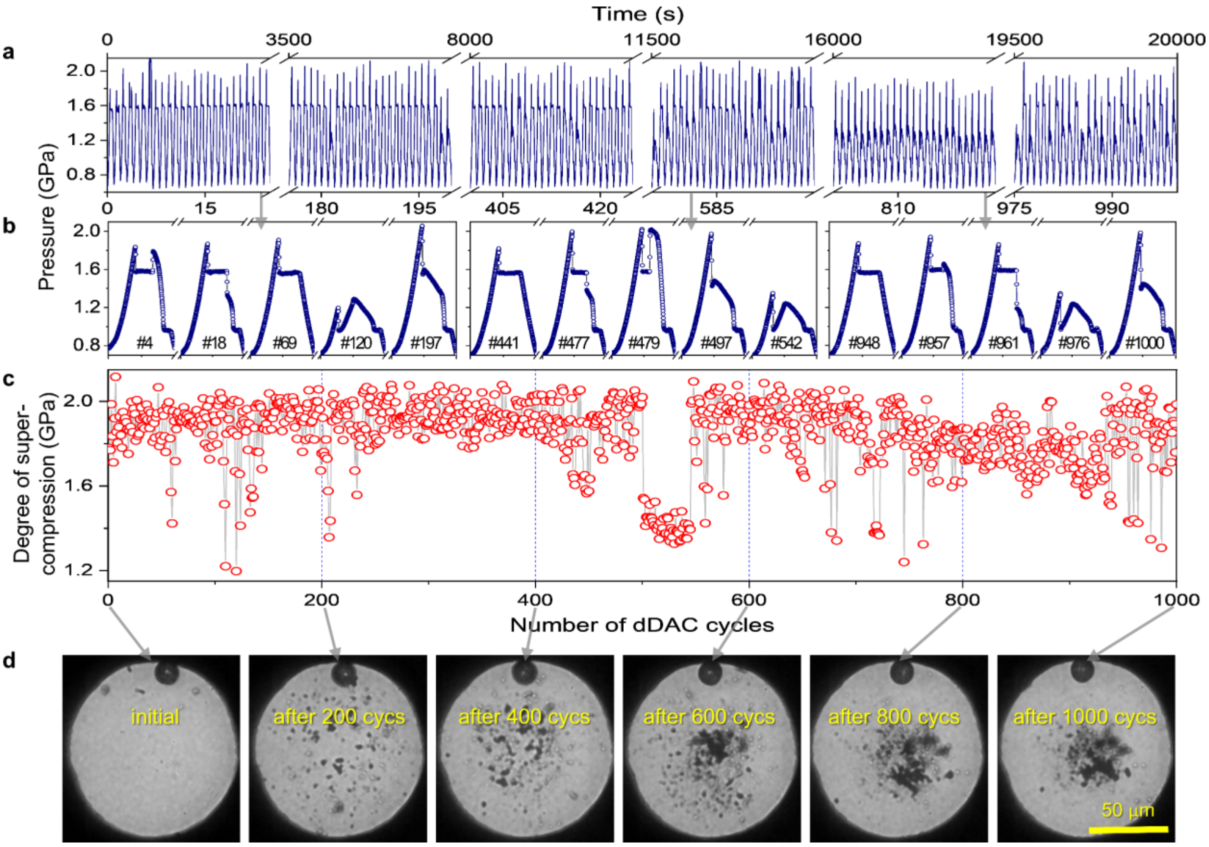

Supplementary Figure 2. Continuous compression and decompression cycles with one dDAC containing H_2O sample. a, 1000 repetitive cycles are plotted with the number of dDAC cycles and elapsed time. **b**, five types of pressure-time curves appear randomly in the raw data. **c**, maximum pressure of SW just before crystallization, assuring the repeatable and reproducible operation of the dDAC. **d**, Soundness of the gasket hole under the massive accumulation of (de)compression cycles reflects no significant change in the appearance of P-t types during the entire experiments.

4. Time-resolved optical images of two crystallization events in Type 2

In Type 2, SW freezes rapidly within few milliseconds (see Fig. 2b and Supplementary Fig. 3). The fast crystallization event is monitored with a high-speed camera; the recording rate is 50000 frames per second and each frame corresponds to 20 μs. Optical images reveal two crystallization events during the single pressure-drop upon crystallization in Supplementary Fig. 3. The first crystallization is completed within 40 μs and the second crystallization subsequently starts from the boundary of the gasket hole (i.e., sample chamber) toward the center.

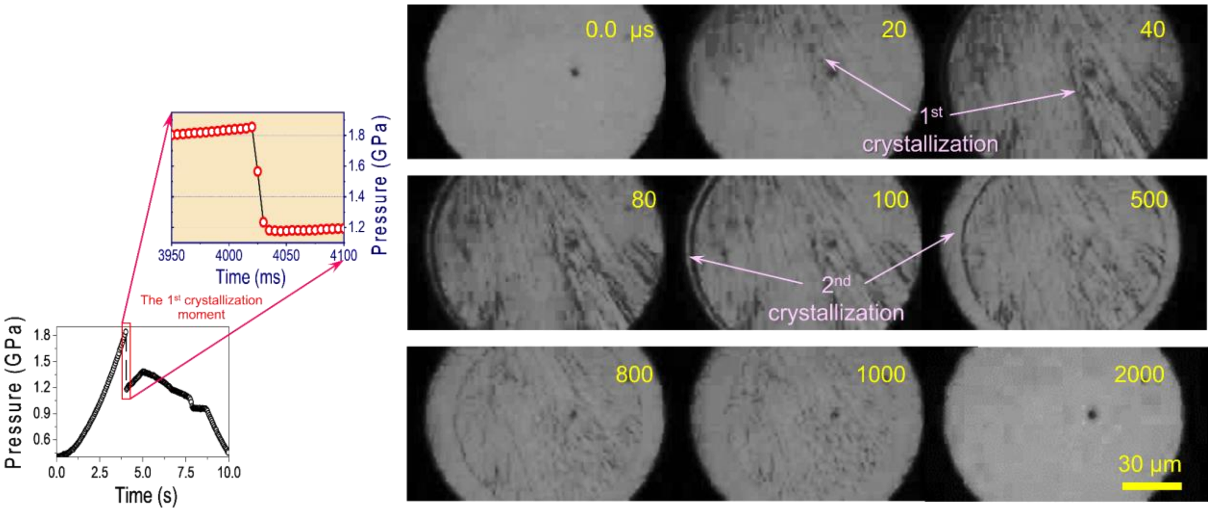

Supplementary Figure 3. Time-resolved micrographs of the crystallization events in Type 2. The recording rate is 50000 frames per second and each frame corresponds to 20 μs. While the first crystallization is completed within two frames (or ~40 μs) in the downward direction, the second transition proceeds from the outer rim to the center within ~2000 μs.

5. In-situ synchrotron X-ray scattering experiment along the pressure-time curves

Phases appeared in the P-t curves are identified by X-ray diffraction experiments which are simultaneously performed with pressure measurement during the dDAC operation. For this study, we installed the in-line ruby pressure measurement (IRPM) system at the X-ray beamlines (1C-XRS beamline in PAL and P02.2 extreme condition beamline in PETRA III at DESY). This method provides simultaneous and precise pressure measurement since ruby fluorescence gives better pressure resolution than metallic pressure markers at low pressure regime[32]. X-ray scattering signals are recorded for every 100 ms during the 20 s pressure cycles (Supplementary Fig. 4) and the pressure is simultaneously recorded with the same measuring rate (Supplementary Fig. 4b). From the diffraction patterns, we find that ice VI and ms-ice VII crystals form along the five types of pressure-time curve; in Types 1 and 2, ice VI forms on the crystallization during compression, whereas ms-ice VII is found at 1.6 GPa after crystallization in Types 3 to 5. Moreover, in Types 4 and 5, the second crystallization upon decompression, indicated by pressure-drop and -jump from 1.6 GPa, results in the formation of ice VI. These results are consistent with the pressure changes in P-t curves. However, we could not resolve the two crystallization events in Type 2 pathway in this synchrotron XRD study, due to insufficient X-ray flux and relatively slow detection. Interestingly, we find unidentified crystal peaks that appears just before the formation of ms-ice VII in the last cycle (marked by a dotted circle). This is the indication of ice XXI phase,

which is shown in main text and in Sections 9 and 10 in Supplementary Information (see Figs. 3e,f in the main text).

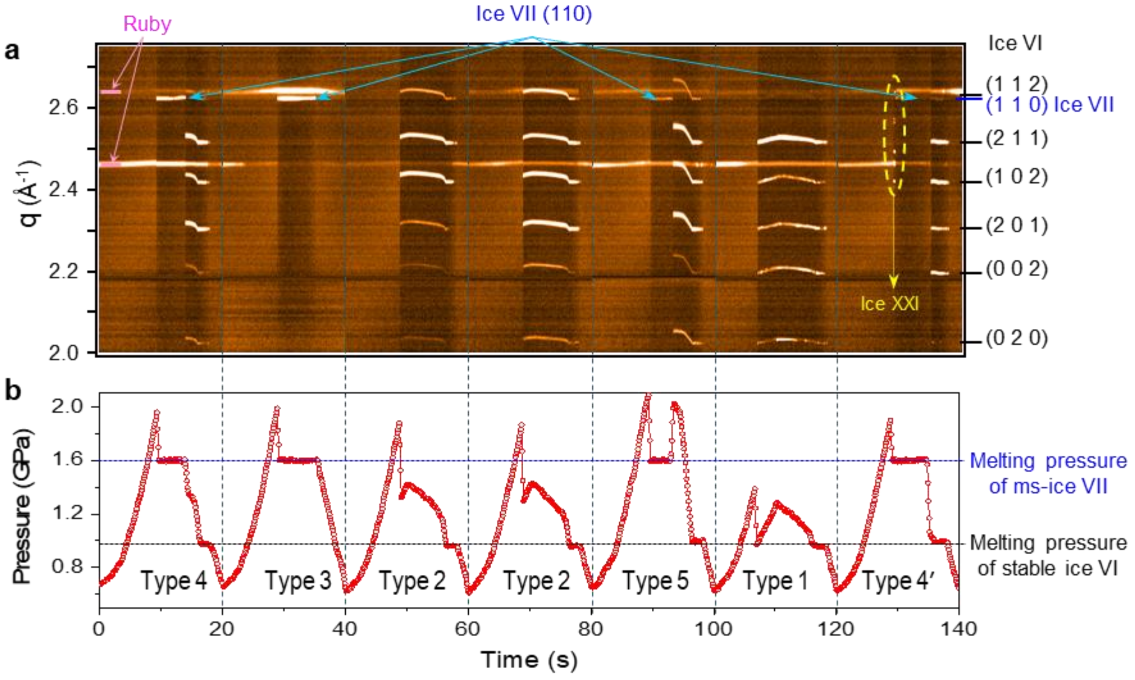

Supplementary Figure 4. X-ray diffraction results recorded simultaneously with pressure. a, In-situ X-ray diffraction patterns are simultaneously measured with **b,** the pressure-time curves. Despite the occurrence of five complex P-t types, only two crystalline phases, ice VI and ms-ice VII, are identified except for the ice XXI phase that directly crystalized from SW in the last cycle.

6. In-situ micro-Raman spectroscopic study along the pressure-time curves

We also measure Raman spectra of the phases appeared during crystallization-melting process. We use parallel, split laser beams having the wavelength of 532 nm to excite both ruby particle and sample, which allow us to simultaneously measure pressure and Raman spectra[31,32]; each Raman spectrum is recorded for every two seconds, but ruby fluorescence signal for pressure is measured for every five ms. Note that the five types of pressure-time curves occur statistically in a wide range of (de)compression periods (i.e., between tens of milliseconds and hundreds of seconds). We identify all phases which appear along the P-t curves in this study (Supplementary Fig. 5); only ice VI phase on crystallization in Types 1 and 2 and the mixture of ms-ice VII and liquid water formed at 1.6 GPa in Types 3 to 5. In case of Type 3, after the plateau at 1.6 GPa on decompression, only liquid water is shown (Supplementary Fig. 5c), which is consistent with the XRD results

(Supplementary Fig. 4). Supplementary Figs. 5d,e show the formation of ice VI from the mixture of ms-ice VII and liquid water at 1.6 GPa on decompression in Types 4 and 5 (i.e., after pressure-drop and -jump from 1.6 GPa). Phase identification along the pressure-time curves is consistent with XRD results in Supplementary Fig. 4. Typical Raman spectra from SW, ice VI and ms-ice VII are compared in Supplementary Fig. 5f.

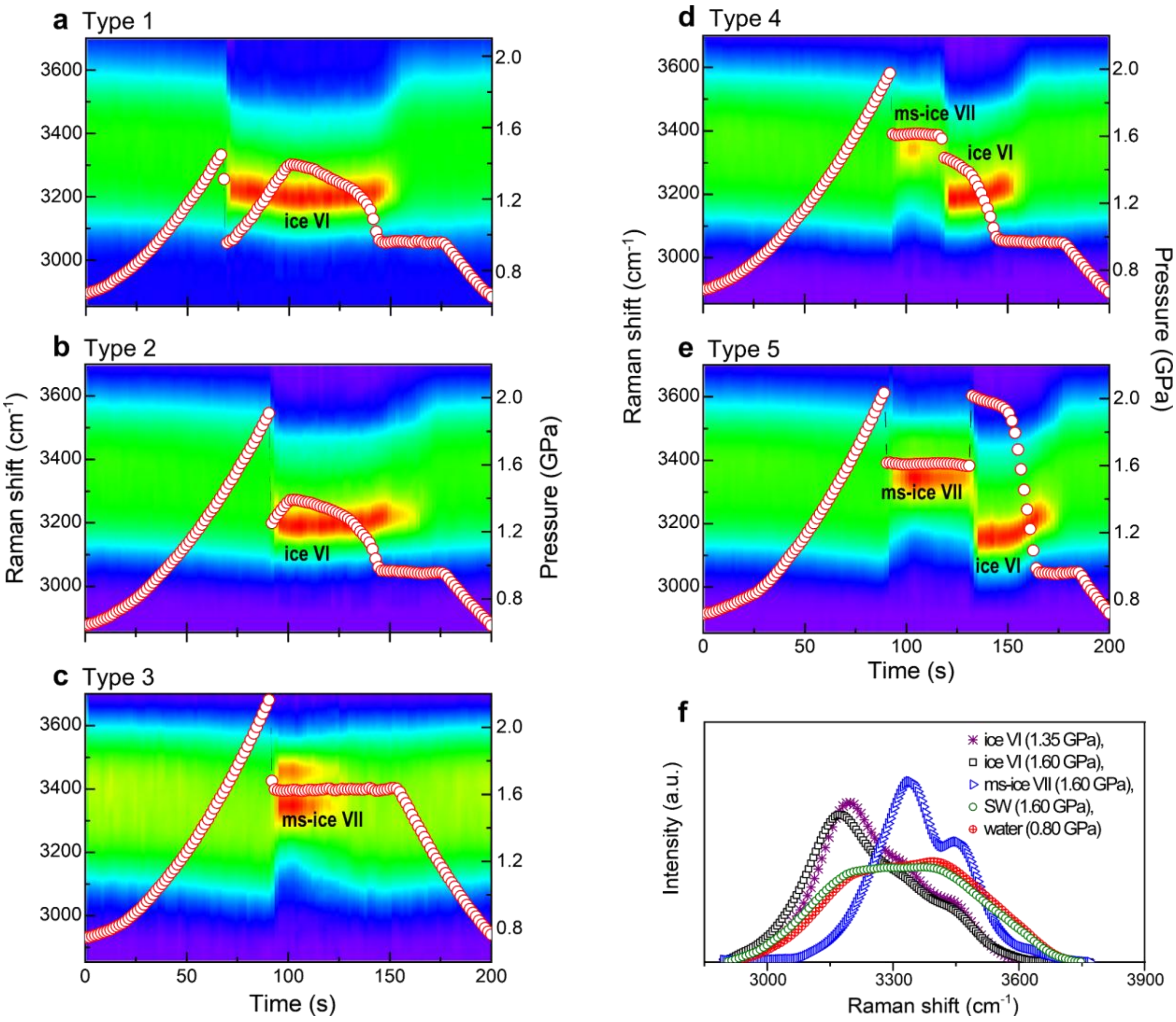

Supplementary Figure 5. Phase identification with Raman spectra along the five types of P-t curve on crystallization-melting process. a-b, Ice VI form after the pressure drops in Types 1 and 2. **c-e,** Ms-ice VII appears after the first pressure drops in Types 3 to 5. **d-e,** The ms-ice VII and water at 1.6 GPa abruptly changes into ice VI phase on decompression, accompanied by the second pressure-drop and -jump in Types 4 and 5, respectively. **f,** Representative Raman spectra of ice VI, ms-ice VII, SW, and liquid water. Excitation laser is localized to only ms-ice VII crystals or SW in the mixture to get their Raman spectra at 1.6 GPa.

7. Synchronization of the X-ray pulse trains and crystallization events during rapid compression in XFEL experiment

To identify the phases that emerged in Type 2, the crystallization events must be matched to the injected X-ray pulse trains. This is a very challenging task, since crystal nucleation events occur statistically, depend on the degree of supercompression, and may have different incubation times with the supercompression level. Thus, if the X-ray pulse train is not properly synchronized with the dDAC operation time, the crystallization events cannot be detected.

In order to predict the time range over which crystallization events occur, we, in advance, have tested the dependence of crystallization time on compression rate and degree of supercompression. Supplementary Fig. 6 shows the five types of P-t curve with various compression times from 0.01 to 10 s. Note that except Type 1, we can observe all types of P-t curves from slow compression of 10 s and fast compression of 10 ms. This means that the compression rates do not significantly affect the crystallization events within this compression range. We find that the crystallization events occur at 8.29±0.15 s for 10 s compression and shift to 8.69±0.23 ms for 10 ms compression. Although the time for crystallization events increases slightly with compression rates, the overall crystallization events can be detected with a narrow time window of 8.5±1.0 ms. Thus, we place the long X-ray pulse trains over a compression time from 7.5 to 9.5 ms at 0.5 ms intervals for the detection of crystallization events. With this experimental scheme, we could successfully synchronize the crystallization events and X-ray pulse trains during compression. In this study, we design that decompression takes one second to check the freezing-melting types of P-t curves with the X-ray diffraction patterns (Fig. 3a and Supplementary Fig. 6d).

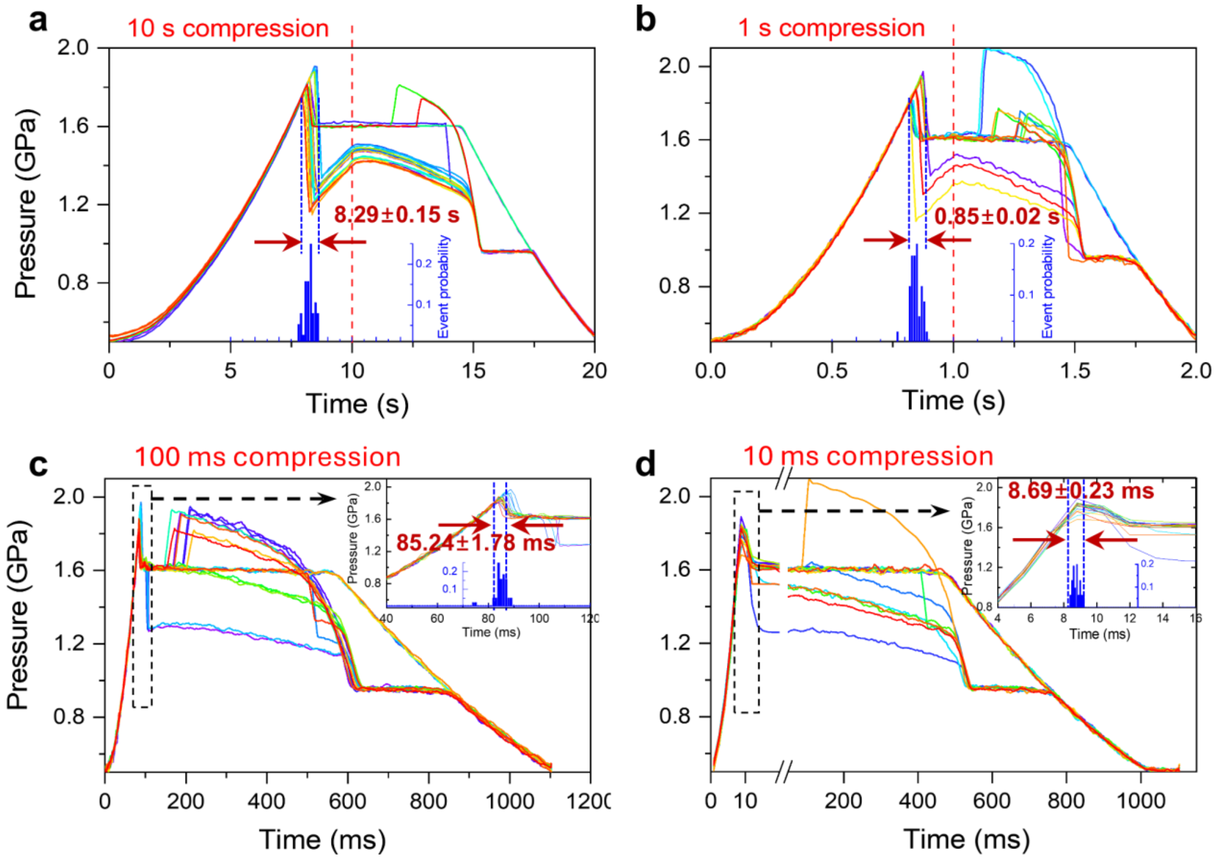

Supplementary Figure 6. **Occurrence of crystallization during compression with different cycle periods.** Regardless of compression rates and shapes of (de)compression wave function, crystallization events happen at similar timescales from 10 s, **a** to 10 ms, **d** if the crystallization time is normalized with the compression time. All compression rates in **a-d** show the typical five types of P-t curves.

8. Installation of dDAC in XFEL and event-synchronizing scheme

Three dDACs are placed in a revolver wheel which is installed in the IC2 vacuum chamber (10^{-4} mbar, HED beamline at EuXFEL) (Supplementary Fig. 7a) [33,34]. The X-ray beam and the sample inside of gasket hole are aligned with the camera system of the IC2 chamber. Piezoelectric actuators operating the dDACs are connected via electrical lines to the voltage amplifier and the power supply located outside the chamber. Two detectors of AGIPD and Varex are installed outside of the IC2 chamber (see Supplementary Fig. 7a). Cr_2O_3 powder is used as a reference material for experimental setup and fine alignment. Detailed procedures on calibration can be found in a previous study[33,34]. A triangular voltage waveform applied to the dDAC for compression and decompression is shifted with respect to the arrival of the 1st X-ray pulse train (See Fig. 3a in the main text).

AGIPD detector with the 0.56 MHz recording rate[35] is used to resolve the event of the first rapid crystallization. Varex detector with the 10 Hz repetition rate is applied for relatively slow transitions of the H_2O phases on decompression. Supplementary Fig. 7b shows XRD patterns which is taken by AGIPD detector during the fast transition. The metastable ice XXI (ms-ice XXI) formed from SW transforms into ms-ice VII. The two crystalline phases coexist for ~50 μs during the transition. The Varex data shows that ms-ice XXI changes into ms-ice VII and finally ice VI on decompression. The broad, diffused scattering intensity signals liquid water coexisting with ms-ice XXI and ms-ice VII. Furthermore, the water signal that is still shown before and after transition reflects that ms-ice XXI only transforms into ms-ice VII and the remained water doesn't (Supplementary Fig. 7b).

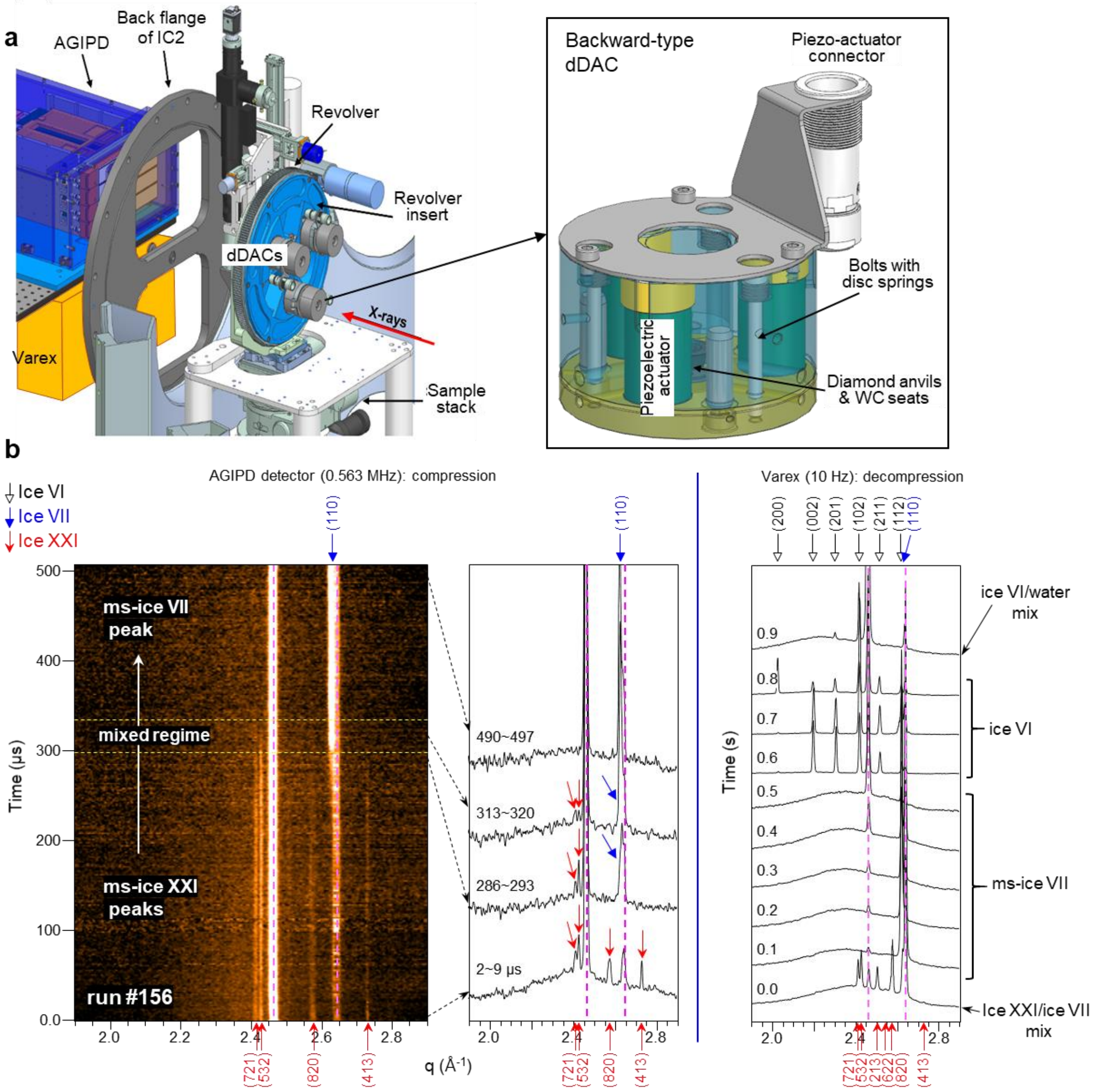

Supplementary Figure 7. dDAC setup for XFEL experiments and time-resolved diffraction data on crystallization events. **a,** Schematic diagram for the installation of vacuum-compatible dDACs on the revolver wheel inside of the IC2 at HED beamline. Three LLNL-type dDACs[31] are replaced with the backward-type dDACs. Two detectors of AGIPD and Varex are located close to the IC2 chamber. **b,** Typical data for time-resolved observations for phase formation and transition detected with both AGIPD and VAREX detectors. The diffraction peaks of the ice XXI are indexed with red arrows at the bottom of the **b** panels based on the identified body-centred tetragonal symmetry.

9. Comparison of the discovered ice XXI with 24 known ice phases

We discover a metastable ice phase, appearing directly from SW on compression (Fig. 3 and Supplementary Fig. 7b). We compare the XRD patterns of the discovered ice phase with those of experimentally obtained ice polymorphs from ice I_h to ice XX[2-6,56-70] and theoretically predicted three metastable ices[23,27,28] (Supplementary Fig. 8a); diffraction patterns of both ice VI and ice VII at 1.6 GPa are experimentally measured in this study or extrapolated based on EOS information[62,63]. Ice XVIII is refined based on shock compression data[2]. Ices T and Y are simulated at high pressures of 5-6 GPa[23,28]. None of the known ice phases match the discovered ice phase, although the direct comparison may not be proper to some of XRD patterns due to different pressure and temperature conditions[37,56-61,64,66,68-70]. For this comparison, all diffraction profiles are reconstructed assuming the same X-ray wavelength of 0.688 Å. Therefore, we conclude that the ice phase discovered is still unknown and can be called ice XXI.

In Supplementary Fig. 8b, many X-ray patterns of the ice XXI with the AGIPD are superimposed resulting in the representative diffraction pattern in Supplementary Fig. 8a. While run numbers 89, 158, and 159 show the eight representative peaks of the ice XXI shown in Supplementary Fig. 8a, other run numbers have missing peaks and different intensity of the peaks. This reflects the development of preferred crystal orientation during the crystallization process.

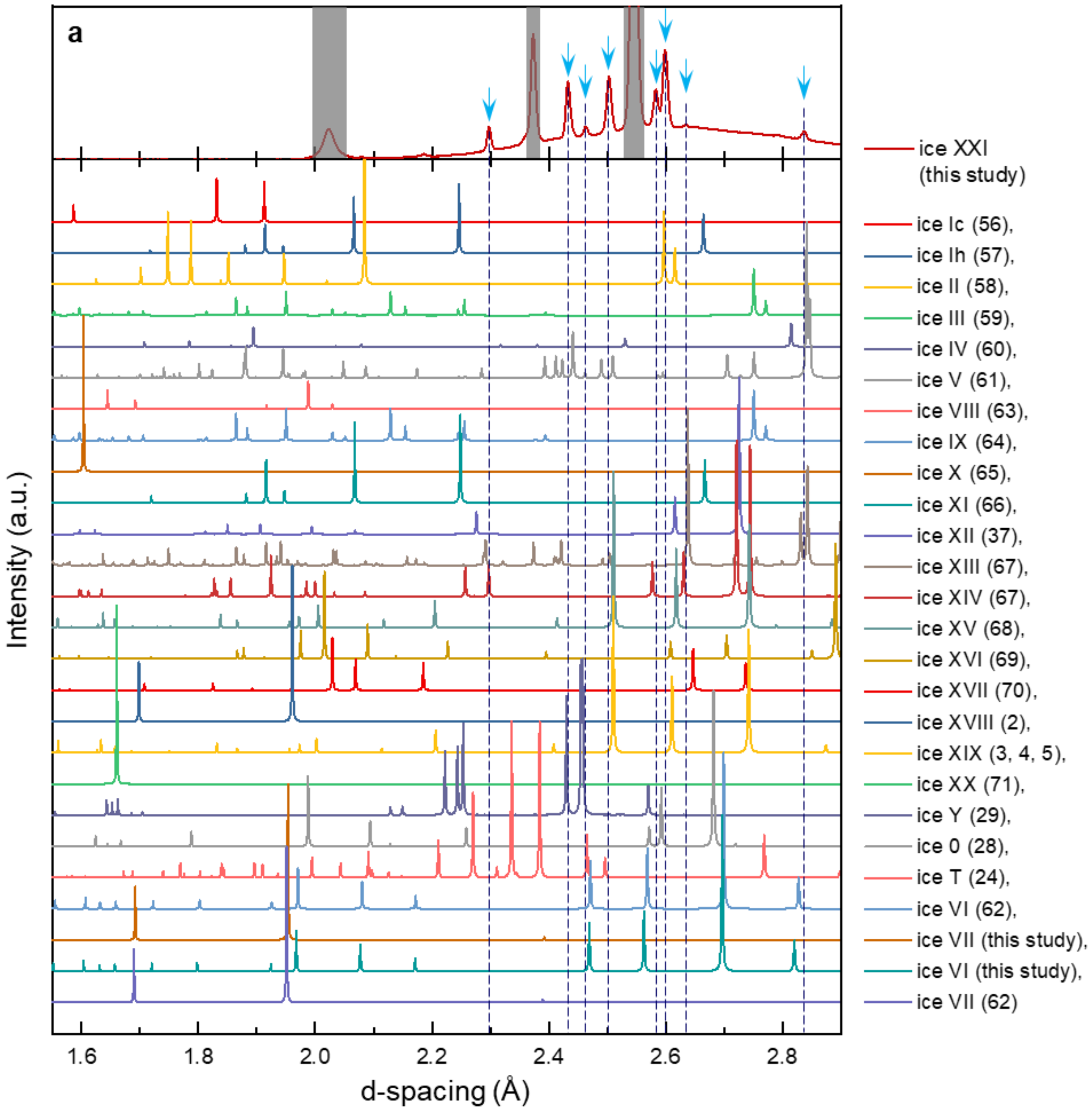

a
Intensity (a.u.)
d-spacing (Å)
1.6
1.8
2.0
2.2
2.4
2.6
2.8
ice XXI (this study)
ice Ic (56),
ice Ih (57),
ice II (58),
ice III (59),
ice IV (60),
ice V (61),
ice VIII (63),
ice IX (64),
ice X (65),
ice XI (66),
ice XII (37),
ice XIII (67),
ice XIV (67),
ice XV (68),
ice XVI (69),
ice XVII (70),
ice XVIII (2),
ice XIX (3, 4, 5),
ice XX (71),
ice Y (29),
ice 0 (28),
ice T (24),
ice VI (62),
ice VII (this study),
ice VI (this study),
ice VII (62)

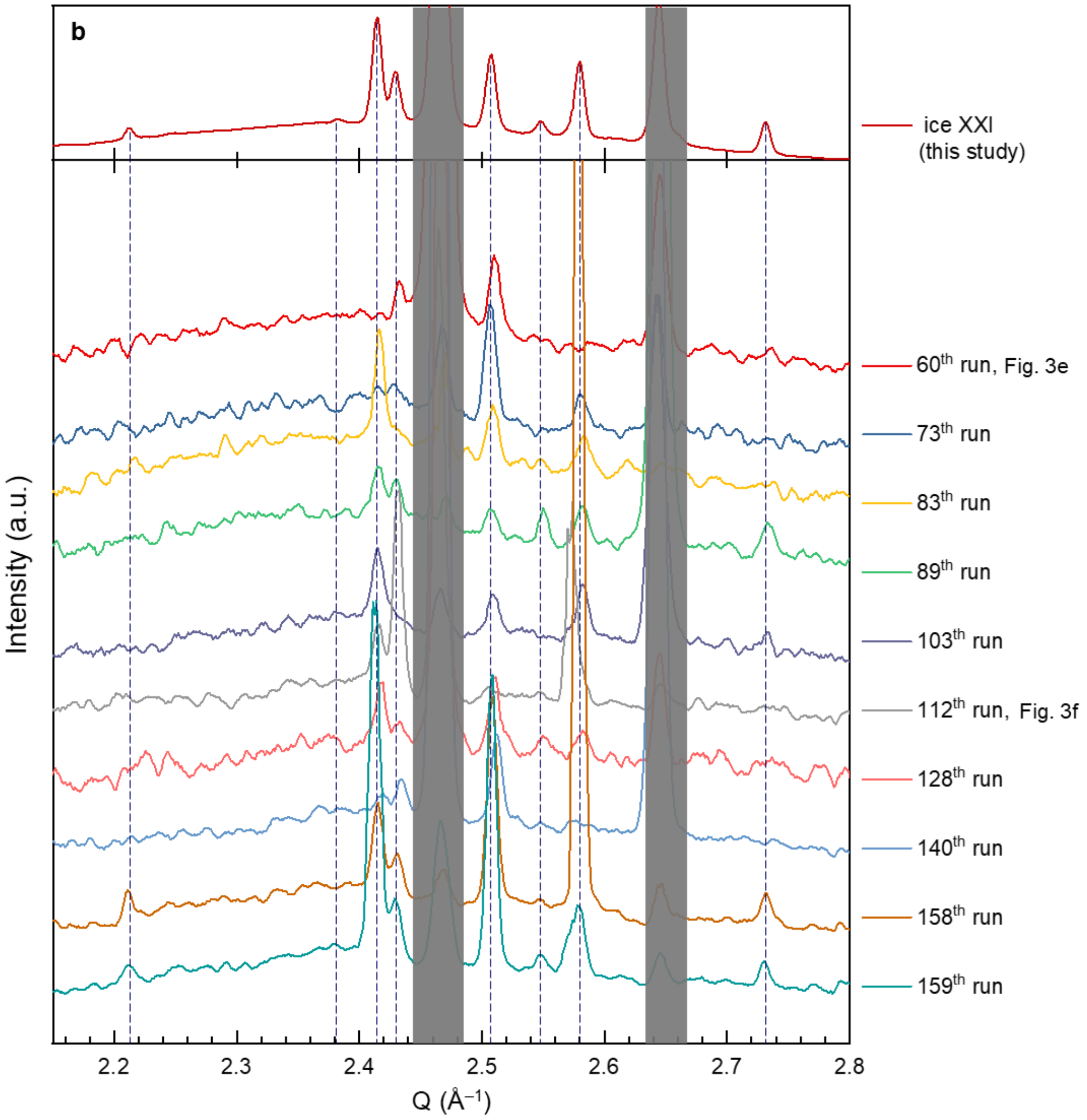

Supplementary Figure 8. Comparison of X-ray diffraction patterns of the discovered ice with 24 stable and metastable ice phases. a, Diffraction peaks of the discovered ice phase do not match with those of the known ice phases. Grey shadow in the diffraction patterns of the discovered ice shows noise signals from ruby and steel gasket. **b,** Raw X-ray patterns from several dDAC-XFEL runs are overlapped and compared with the representative diffraction patterns in **a**.

10. Structural identification of the ice XXI based on single-crystal X-ray diffraction study

Although ms-ice XXI typically appears shortly, we find that ice XXI coexists with SW as shown in Supplementary Figure 7b. This means that ice XXI may persist longer than a few hundred microseconds under a certain condition. Thus, we repeat the crystallization process over a thousand times. While, in most cases, stable

ice VI or metastable ice VII formed, we also often observe ice XXI coexisting with supercompressed water, like the mixture of ms-ice VII and SW at 1.6 GPa. In this stage, we cannot clearly specify the condition making the mixture of the ice XXI and SW. Once the polycrystalline ice XXI is formed in SW, we carefully adjust the voltage of the piezoelectric actuator to melt small crystals and finally get a small single crystal of the ice XXI (See Supplementary Figure 9a). Then, we slowly compress dDAC to grow the crystal and fill the gasket hole with the single crystalline, ice XXI large enough for X-ray diffraction (Supplementary Fig. 9b).

We carry out single-crystal X-ray diffractions to understand structure of the ice XXI (P02.2 extreme condition beamline in PETRA III at DESY). Wavelength and beam size of X-rays are 0.2896 Å and 7.5 (w) × 1.7 (h) μm^2, respectively. Diffraction images are collected using Perkin Elmer detector. Diffraction pattern for each single crystal is measured with a 1-s exposure and it is repeated for a series of ω scans over a range of ±11° at 0.5° intervals. Furthermore, the representative diffraction pattern of a single-crystal ice XXI is prepared by accumulating diffraction data for successive 20° oscillations over a 10 s exposure time (Supplementary Fig. 9c). The XRD data collection is repeated for seven independently grown single crystals of ice XXI with different crystallographic orientations.

The space group and unit cell parameters are refined from the measured XRD images using the CrysAlisPro™ program (Supplementary Table 1). The data are scaled and merged, and the space group of ice XXI is determined to be body-centred tetragonal structure with the unit cell dimensions of $a = b = 20.197$ Å and $c = 7.891$ Å at approximately 1.6 GPa. All observed Bragg peaks satisfy the extinction conditions consistent with the extinction symbol $I{-}{-}d$ which imposes the following selection rules; hkl: $h + k + l = 2n$, $hk0$: $h + k = 2n$, $0kl$: $k + l = 2n$, hhl: $2h + l = 4n$, $00l$: $l = 4n$, $h00$: $h = 2n$, and $h\bar{h}0$: $h = 2n$. These extinction rules are consistent with two space groups of $I\bar{4}2d$ and $I4_1md$. We minimize the merging R-factor (R_{int}) and eventually assign the space group $I\bar{4}2d$ to ice XXI. This space group ($I\bar{4}2d$) is well fit for the polycrystalline diffraction data of ice XXI with unit cell parameters of $a = b = 20.085$ Å and $c = 7.828$ Å obtained during crystallization in XFEL experiment (Fig. 3g in the main text or Supplementary Fig. 9d).

Using the determined space group and lattice parameters, we solve the crystal structure of ice XXI by intrinsic phasing with the ShelXT structure solution program and refine it through least-square minimization with ShelXL refinement program integrated within the Olex2 software package[71,72] (Supplementary Table 1).

Only oxygen atoms can be refined from this X-ray diffraction dataset. The final refined model contains ten crystallographically distinct oxygen sites in the asymmetric unit (Supplementary Table 2). Nine of these occupy general 16-fold positions and one lies on an 8-fold symmetry position of the space group $I\bar{4}2d$,

resulting in a total 152 molecules per unit cell. Refinement with isotropic temperature factors converges to an R_1 = 0.094 for the 427 observed reflections ($I > 2\sigma(I)$) and $R_1 = 0.128$ for all data (Supplementary Table 1). The goodness-of-fit is 1.328, and the largest residual peaks in the difference map are on the order of 0.3 e/Å^3, indicating a clean solution. Given the inherent difficulties associated with high-pressure data collection and the complexity of this structure, this refinement statistics are considered acceptable.

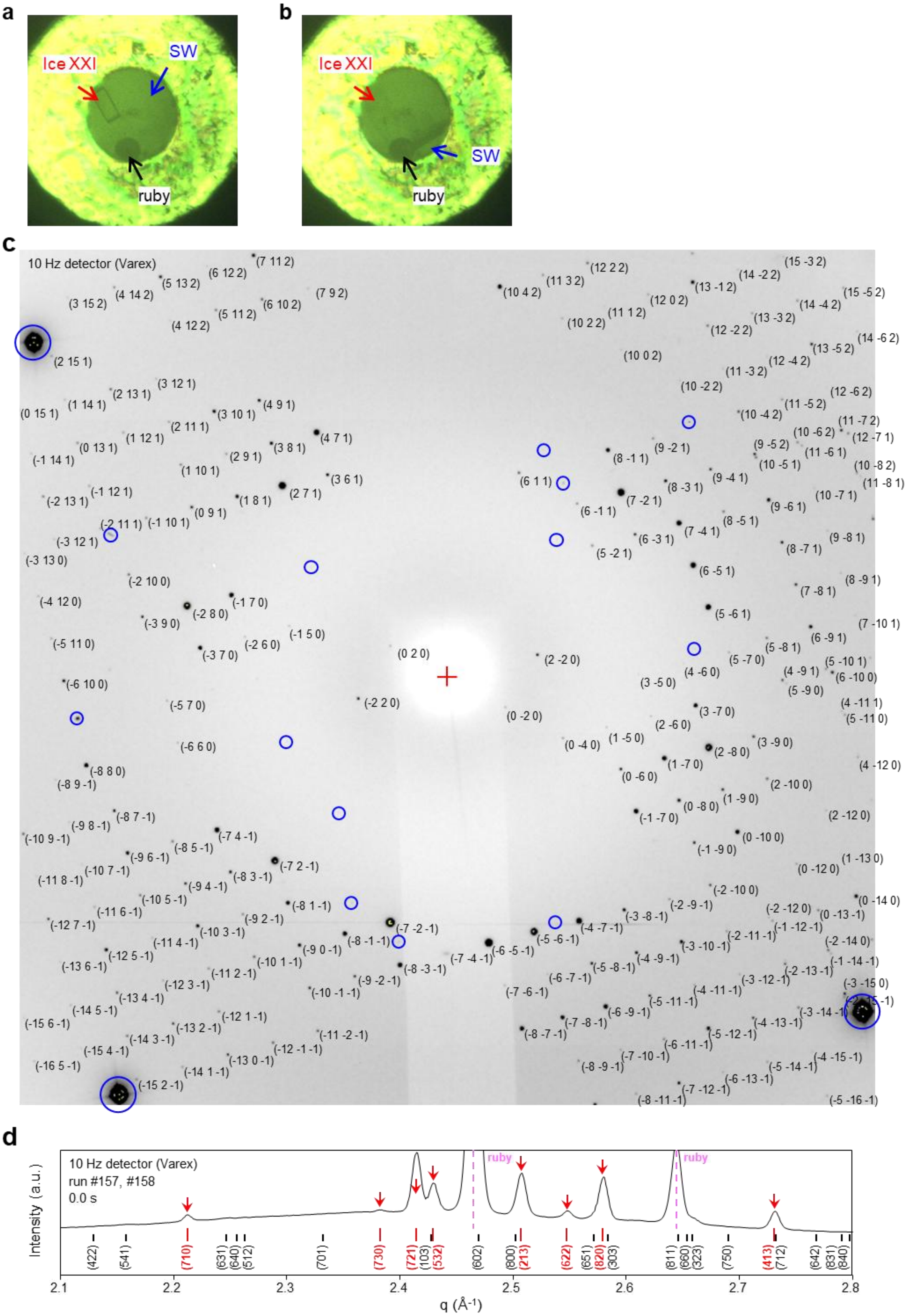
a
Ice XXI
SW
ruby
b
Ice XXI
SW
ruby
c
10 Hz detector (Varex)
d
10 Hz detector (Varex)
run #157, #158
0.0 s
ruby
ruby
Intensity (a.u.)
(422)
(541)
(710)
(631)
(640)
(512)
(701)
(730)
(721)
(103)
(532)
(602)
(800)
(213)
(622)
(651)
(820)
(303)
(811)
(660)
(323)
(750)
(413)
(712)
(642)
(831)
(840)
2.1
2.2
2.3
2.4
2.5
2.6
2.7
2.8
q (Å^{-1})

Supplementary Figure 9. A single crystal of ice XXI grown in the dDAC and representative XRD spot pattern indexed with the body-centred tetragonal structure. a, A small crystal of the ice XXI formed through nucleation and slow melting process and **b,** a large single crystal grown from the initial crystal. **c,** Representative single-crystal XRD spots of ice XXI are indexed with the refined ice XXI structure, $I\bar{4}2d$. Blue circles come from diamonds. **d,** 1D diffraction profile of ice XXI obtained from XFEL experiment, indexed with the most well-fitted body-centred tetragonal structure.

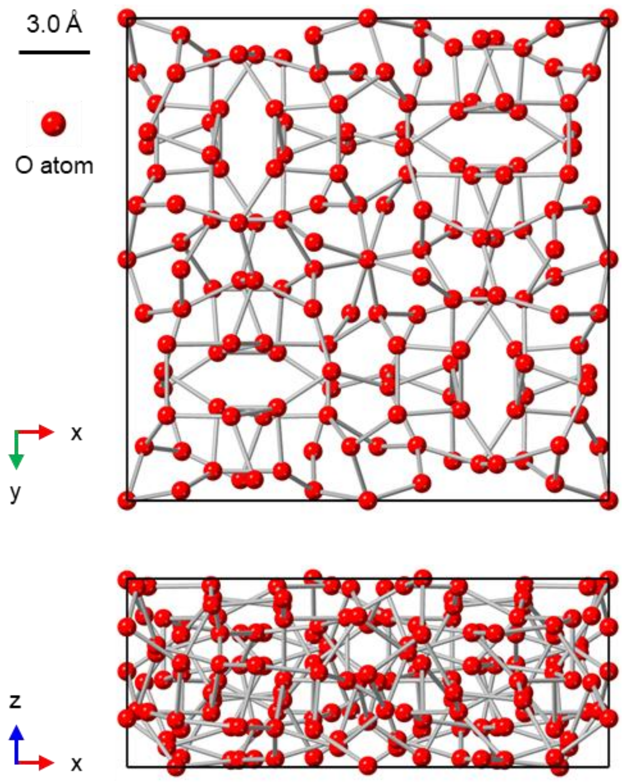

Supplementary Figure 10. Crystal structure of ice XXI. Only oxygen atoms are refined; bonds between oxygen atoms are displayed for atom pairs separated by less than 3.0 Å, resulting in exactly four bonds for every oxygen atom.

Supplementary Table 1. Crystallographic data collection and structural refinement for the merged diffraction data of single-crystal ice XXI under 1.6 GPa and room temperature.

	Ice XXI
**Crystal data**	
Space group	$I\bar{4}2d$
Unit cell parameters	
a (Å)	20.1966(16)
c (Å)	7.8912(6)
Volume (Å^3)	3218.8(6)
**Data collection**	
Radiation source	PETRA P02.2
Wavelength (Å)	0.2896
Detector	Perkin Elmer detector
Pressure (GPa)	1.6
Temperature (K)	Room temperature
Total oscillation angle (°)	23
Number of images	46
Exposure time per image (s)	1
Oscillation angle per image (°)	0.5
Resolution (Å)	1.0
Max angle (°)	16.65
**Data reduction**	
Range of reflections	$-20 \leq h \leq 20$, $-20 \leq k \leq 19$, $-7 \leq l \leq 7$
Number of reflections	4726
Number of unique reflections	819
Number of used reflections ($I > 2\sigma(I)$)	427
Completeness (%)	97.38
Redundancy	5.929
I/σ(I)	10.46
R_{int}	0.2217
**Data refinement**	
Number of parameters	39
R_1, all data	0.1275
R_1, $I > 2\sigma(I)$	0.0941
wR_2, all data	0.3422
wR_2, $I > 2\sigma(I)$	0.2678
Goodness-of-fit on F^2	1.328
Largest diffraction peak/hole	0.32 / –0.36
Number of oxygen refined per unit cell	152
Number of H_2O per unit cell	152
Density calculated from refined (g/cm^3)	1.413

Supplementary Table 2. Structural parameters of ice XXI under 1.6 GPa and room temperature.

Site	Fractional coordinates			U_{iso}	Multiplicity
	x	y	z		
O1	0	0.5	0.5073(16)	0.066(3)	8
O2	0.8980(3)	0.6141(4)	0.0006(9)	0.065(3)	16
O3	0.8236(4)	0.9173(4)	0.8562(10)	0.066(3)	16
O4	0.5611(3)	0.6782(4)	0.1457(10)	0.065(3)	16
O5	0.7678(3)	0.5754(3)	0.0379(9)	0.061(3)	16
O6	0.9566(4)	0.7359(4)	0.9584(9)	0.067(3)	16
O7	0.8881(3)	0.5357(4)	0.6983(9)	0.063(3)	16
O8	0.0205(4)	0.3879(4)	0.2985(10)	0.069(3)	16
O9	0.6928(4)	0.6882(4)	0.0437(9)	0.077(3)	16
O10	0.8257(4)	0.7838(4)	0.9630(9)	0.076(3)	16

The space group is $I\bar{4}2d$ with a = 20.1966(16) Å and c = 7.8912(6) Å.

11. Molecular dynamics study of supercompressed water by TIP4P/ICE model

The results of MD simulations with SPCfw model are given in Figs. 4d-f. Here, we show the MD results with TIP4P/Ice model, exhibiting the structural changes of SW with pressure. In Supplementary Fig. 10, all results are qualitatively consistent with SPCfw model in the main text (Fig. 4) and in Supplementary Fig. 11. Potential energy and density of the SW show different slopes at approximately 2 GPa. Pair distribution function (PDF) exhibits the evolution of the SW from high-density water (HDW) to very-high-density water (VHDW) from 1.0 to 3.2 GPa, which exhibit distinctly enhancing shoulder of the first peak at approximately 3.3 Å and of the second peak at approximately 4.8 Å. The representative angles of HDW is shown in Supplementary Figs. 10e,f. As pressure increases, the second nearest molecules (marked by blue color in Supplementary Fig. 10e) place at the interstitial position between the first nearest molecules in HDW. Thus, the population of the angles with 48° decreases and that for 65° increases with pressure. When pressure increases further, the two angles merge together, which is the characteristics of VHDW. In this case, the water molecules in the second nearest molecules (marked by blue color) lose the hydrogen bonding with the water molecules in the first nearest molecules, resulting in distorted hydrogen bonds. Angle distribution functions (ADF) of $O'_{1\text{–}4}$-O-$O''_{5\text{–}8}$ and O-H-O_2^H are depicted in Supplementary Figs. 10g,h. Here, $O'_{1\text{–}4}$-O-$O''_{5\text{–}8}$ is the ADF between the first and the second nearest neighbor oxygen atoms. O_2^H is the O atom of water molecule that is included in a sphere with radius of 3.5 Å from the O atom of the central molecule.

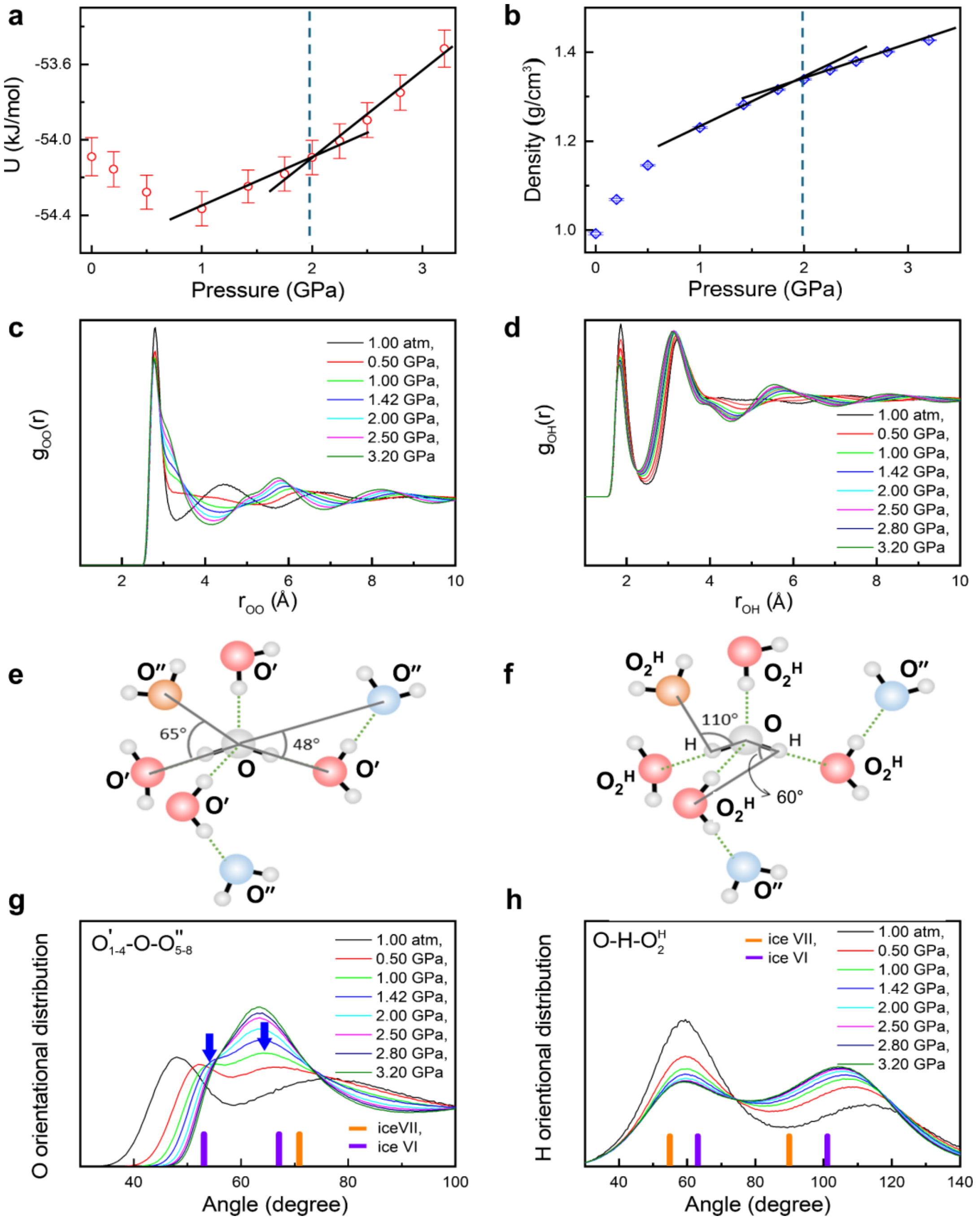

Supplementary Figure 11. Simulation results with TIP4P/ICE model. a, b, show total energy and density with pressure, respectively. Data are presented as mean values, with error bars indicating the standard deviation calculated from 500 data points sampled every 10 ps over trajectories of 15–20 ns. **c, d,** show pair distribution functions (PDFs) of oxygen-oxygen and oxygen-hydrogen, respectively. **e, f,** show schematic diagram for the local order of HDW with the 1st and 2nd nearest neighboring H_2O molecules. Angles of $O'_{1\text{–}4}$-O-$O''_{5\text{–}8}$ and O-H-O_2^H are defined in a reference[25]. Here, $O'_{1\text{–}4}$ and $O''_{5\text{–}8}$, respectively, are defined as the 1st-4th and 5th-8th nearest neighboring O atoms of the O atom of the central water molecule. **g,** Angle distribution of oxygen atoms

in nearest and second nearest neighbors. **h,** Angle distribution function of O-H-O_2^H. and that is included in a sphere with radius of 3.5 Å from the O atom of the central molecule.

12. Pair distribution functions (PDFs) of O-H pairs and angle distribution functions (ADFs) from SPCfw model

In SPCfw model, PDFs (g(r)) of O-H pairs exhibit relatively small change with pressure over 1.0 GPa, compared with those of O-O pairs. Angle distribution function of O-H-O_2^H reflects that the hydrogen bonding resembles somewhat between ice VI-like and ice VII-like structure as pressure increases from 1.0 to 3.2 GPa.

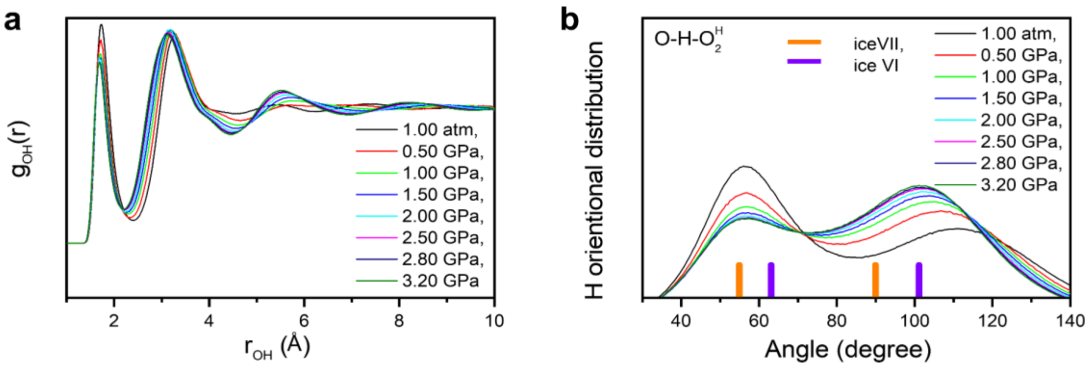

Supplementary Figure 12. Changes in H-O pairs with pressure simulated with SPCfw model. a, b, show g(r) (PDF) of O-H pairs and angle distribution function of O-H-O_2^H with pressure, respectively.

References

55. Wang, L., Cho, Y. C., Lee, Y.-H., Jeon, S. & Lee, G. W. Role of hypercooling limit in supercooling behavior and glass formation. *Phys. Rev. Mater.* **8**, 063401, (2024).

56. Dowell, L. G. & Rinfret, A. P. Low-temperature forms of ice as studied by X-ray diffraction. *Nature* **188**, 1144-1148 (1960).

57. Fortes, A. D. et al. No evidence for large-scale proton ordering in Antarctic ice from powder neutron diffraction. *J. Chem. Phys.* **120**, 11376-11379 (2004).

58. Kamb, B. Ice II. a proton-ordered form of ice. *Acta Cryst.* **17**, 1437-1449 (1964).

59. Kamb, B. & Prakash, A. Structure of ice III. *Acta Cryst.* B **24**, 1317-1327 (1968).

60. Engelhardt, H. & Kamb, B. Structure of ice IV, a metastable high-pressure phase. *J. Chem. Phys.* **75**, 5887-5899 (1981).

61. Kamb, B., Prakash, A. & Knobler, C. Structure of ice. V. *Acta Cryst.* **22**, 706-715 (1967).

62. Bezacier, L. et al. Equations of state of ice VI and ice VII at high pressure and high temperature. *J. Chem. Phys.* **141**, 104505 (2014).

63. Besson, J. M. et al. Variation of interatomic distances in ice VIII to 10 GPa. *Phys. Rev. B* **49**, 12540 (1994).

64. La Placa, S. J., Hamilton, W. C., Kamb, B. & Prakash, A. On a nearly proton-ordered structure for ice IX. *J. Chem. Phys.* **58**, 567-580 (1973).

65. Hemley, R. J. et al. Static compression of H_2O-ice to 128 GPa (1.28 Mbar). *Nature* **330**, 737-740 (1987).

66. Leadbetter, A. J. et al. The equilibrium low-temperature structure of ice. *J. Chem. Phys.* **82**, 424-428 (1985).

67. Salzmann, C. G., Radaelli, P. G., Hallbrucker, A., Mayer, E. & Finney, J. L. The preparation and structures of hydrogen ordered phases of ice. *Science* **311**, 1758-1761 (2006).

68. Salzmann, C. G., Radaelli, P. G., Mayer, E. & Finney, J. L. Ice XV: a new thermodynamically stable phase of ice. *Phys. Rev. Lett.* **103**, 105701 (2009).

69. Falenty, A., Hansen, T. C. & Kuhs, W. F. Formation and properties of ice XVI obtained by emptying a type sII clathrate hydrate. *Nature* **516**, 231-233 (2014).

70. Del Rosso, L., Celli, M. & Ulivi, L. New porous water ice metastable at atmospheric pressure obtained by emptying a hydrogen-filled ice. *Nat. Commun.* **7**, 13394 (2016).

71. Sheldrick, G. M. Crystal structure refinement with SHELXL. *Acta Cryst.* C **71**, 3–8 (2015).

72. Dolomanov, O.V., Bourhis, L.J., Gildea, R.J., Howard, J.A.K., Puschmann, H. OLEX2: A complete structure solution, refinement and analysis program *J. Appl. Cryst.* **42**, 339-341. (2009).
